# Silicon-Bridged Bis(12-crown-4) Ethers as Ionophores for Sodium Ion-Selective Electrodes

**DOI:** 10.3390/molecules30040925

**Published:** 2025-02-17

**Authors:** Shoichi Katsuta, Yoshiyasu Ino, Hiroto Wakabayashi

**Affiliations:** 1 Department of Chemistry, Graduate School of Science, Chiba University, 1-33 Yayoi-cho, Inage, Chiba 263-8522, Japan; 2 Department of Chemistry, Division of Advanced Science and Engineering, Graduate School of Science and Engineering, Chiba University, 1-33 Yayoi-cho, Inage, Chiba 263-8522, Japan

**Keywords:** bis(crown ether), silicon, 12-crown-4, ionophore, sodium ion, ion selective electrode

## Abstract

A new Na^+^ ionophore with two 12-crown-4 moieties on silicon atoms and hydrophobic hydrocarbon groups on silicon atoms has been synthesized. The silicon-bridged bis(12-crown-4)s were easily obtained in high yield by simply mixing dichlorodiorganosilane and 2-hydroxymethyl-12-crown-4 under room temperature and nitrogen atmosphere. Seven compounds with different hydrocarbon substituents were synthesized. To investigate their properties as ionophores, PVC membrane-type ion-selective electrodes incorporating them were prepared, and the ion selectivity coefficients were determined. The typical selectivity sequence is Na^+^ > K^+^ > Rb^+^ > Cs^+^ > NH_4_^+^ > Li^+^ > Ca^2+^ > Mg^2+^ > H^+^. The magnitude of selectivity depends on the structures of hydrocarbon substituents on the silicon atoms. The compound with two 2-ethylhexyl groups has particularly good Na^+^ selectivity, and the performance of the electrode is equal to or better than that of an electrode using a commercially available Na^+^ ionophore, malonate-bridged bis(12-crown-4). The electrode also showed better-aging stability than that of another known Na^+^ ionophore, tetraethyl 4-*tert*-butylcalix[4]arene-*O*,*O′*,*O″*,*O‴*-tetraacetate, indicating high utility.

## 1. Introduction

The concentration of sodium ions (Na^+^) in a living body is closely related to the metabolic reactions of the body and is often used to diagnose various diseases such as hypertension symptoms, renal diseases, and neurological disorders, as well as the concentration of this ion in the body [1,2]. In addition, the analysis of Na^+^ is also important in the fields of drinking water [3] and food control [4].

Traditionally, spectral methods such as flame photometry and chromatographic methods such as ion chromatography have been used to measure Na^+^, but they have sometimes the disadvantages of requiring large and expensive equipment and relatively long measurement times [5,6]. To solve this problem, the potentiometric method using the Na^+^-selective electrodes consisting of a membrane containing ionophores that react selectively with Na^+^ is used to determine Na^+^ in aqueous solutions; the method is faster and less expensive than the former methods [7].

Under these technical conditions, several compounds have been reported that can be used as ionophores for Na^+^-selective electrodes. Representative compounds include 16-crown-5 derivatives [8], 4-*tert*-butylcalix[4]arene derivatives [9,10,11], and bis(12-crown-4) ether derivatives [12,13].

**Figure 1:**
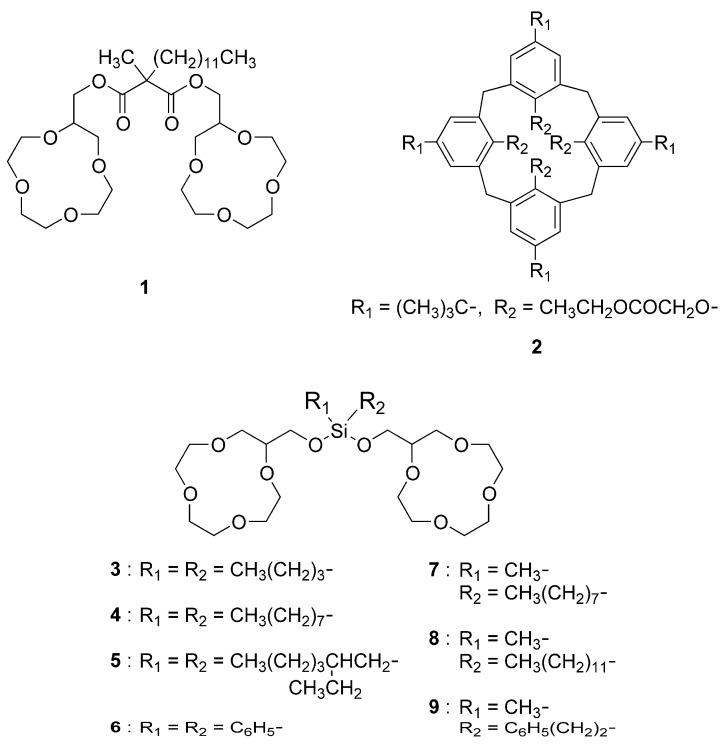
Structural formulas of ionophores used in this study.

Among them, bis[(12-crown-4)methyl]-2-dodecyl-2-methylmalonate (**1** in Figure 1) is a commercially available Na^+^-selective ionophore that forms a stable complex by sandwiching one Na^+^ ion between two crown rings and has excellent Na^+^ selectivity [12,13]. The Na^+^-selective electrodes using polymeric membranes containing **1** are in practical use; the selectivity of **1** between Na^+^ and other ions is generally inferior when compared to the electrode of another commercially available Na^+^-ionophore, tetraethyl 4-*tert*-butylcalix[4]arene-*O*,*O*′,*O*″,*O*‴-tetraacetate (**2** in Figure 1), but is equivalent or even superior in Na^+^-Li^+^, Na^+^-Ca^2+^, and Na^+^-Mg^2+^ selectivity. The ionophores **1** and **2** are, however, expensive and not suitable for large-scale applications such as extractants for Na^+^. In addition, the synthetic raw material of **1**, 2-dodecyl-2-methylmalonic acid is not commercially available, making it difficult to synthesize **1** in general laboratories.

The authors therefore conducted research with the aim of finding new compounds that have excellent ion selectivity, can be used as ionophores, and are still easy to synthesize. In the past, malonic acid derivatives have often been used as cross-linkers in the synthesis of bis(12-crown-4) compounds such as **1**. In this study, we focused on dichlorodiorganosilanes as cross-linkers, which are relatively inexpensive and available in a wide variety of compounds, and synthesized a new type of bis(12-crown-4)s cross-linked with various diorganosilanes (**3**–**9** in Figure 1). In addition, poly (vinyl chloride) (PVC) membrane ion-selective electrodes were prepared using the synthesized ionophores as sensitizers, and the potential responses of the electrodes to Na^+^ in water, in the presence of other cations (Li^+^, K^+^, Rb^+^, Cs^+^, Mg^2+^, Ca^2+^, NH_4_^+^, and H^+^), were investigated. The selectivity was compared with that of the ion-selective electrodes using known Na^+^ ionophores **1** and **2**. The effect of the structure of the two hydrocarbon substituents on the silicon atom upon the ion selectivity was also discussed. Note that the compound **7** was only synthesized and not evaluated for ion selectivity due to its structural similarity to **8**.

## 2. Results and Discussion

### 2.1. Synthesis of Ionophores

All the silicon-bridged bis(12-crown-4) compounds were obtained as liquids with a good yield (about 80−90%) by the following reaction of dichlorodiorganosilane (R_1_R_2_SiCl_2_) with 2-hydroxymethyl-12-crown-4 (12C4-CH_2_OH).

R_1_R_2_SiCl_2_ + 12C4 − CH_2_OH → R_1_R_2_Si (OCH_2_ − 12C4)_2_ + 2 HCl. (1)

The operation is only to mix R_1_R_2_SiCl_2_ and 12C4-CH_2_OH in a mole ratio of 1: *x* (*x* = 2.1–2.4) at room temperature. The reason for adding a small excess of 12C4-CH_2_OH relative to the double equivalent of R_1_R_2_SiCl_2_ is to ensure that only the hydrophilic 12C4-CH_2_OH remains as unreacted raw material to facilitate isolation of the hydrophobic target product by toluene extraction. Since both the raw materials and the product are liquids, no solvent was needed for the reaction. According to the mass spectra of the products (see Supplementary Materials), a small amount of the 2:2 reactant, 12C4-CH_2_O-SiR_1_R_2_-O-SiR_1_R_2_-OCH_2_-12C4, was always observed as a by-product. However, according to the integration values of the ^1^H NMR signals, the number ratio of 12C4-CH_2_O to R_1_R_2_Si is almost 2 (1.8–2.0), suggesting that the proportion of the by-product was small. The purity of the products based on the peak area of the chromatograms of GC-FID was 86%, 95%, 88%, 98%, 94%, 100%, and 99% for **3**, **4**, **5**, **6**, **7**, **8**, and **9**, respectively.

### 2.2. Potential Responses of PVC Membrane Ion-Selective Electrodes with Synthesized Ionophores

**Figure 2:**
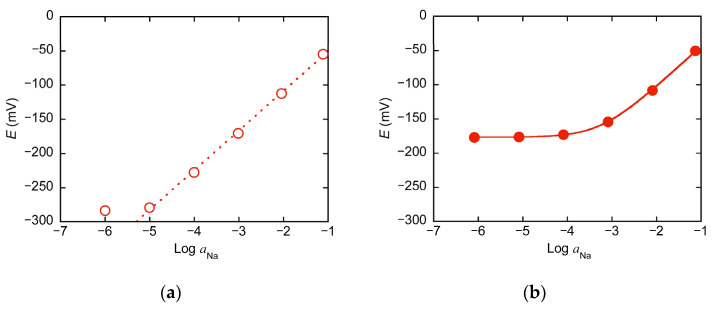
Dependence of emf (*E*) of the electrode containing **5** on the logarithmic value of activity of Na^+^ (log *a*_Na_) in the aqueous NaCl solution at 25 °C (**a**) and that in the presence of 0.050 mol/L KCl (**b**). The dashed line in (**a**) shows the linear regression line for log *a*_Na_ values ranging from −5 to –1. The solid line in (**b**) shows the nonlinear regression curve based on Equation (3).

PVC membrane ion-selective electrodes were prepared using the ionophores except **7**. The emf (*E*) of each electrode was measured when it was immersed in aqueous solutions containing different concentrations of NaCl together with a double-junction type Ag-AgCl reference electrode. For electrodes with any ionophore, *E* showed a similar dependence on the logarithm of the Na^+^ activity (*a*_Na_). As an example, Figure 2a shows the relationship between *E* and log *a*_Na_ for a PVC ion-selective electrode prepared with **5**, where the Na^+^ activity was calculated using the extended Debye-Hückel equation (ion size parameter for Na^+^ = 4 Å [14] was used). As can be seen from Figure 2a, *E* increases with increasing log *a*_Na_. The slope is moderate for log *a*_Na_ between −6 and −5 but increases and becomes constant (58 mV/decade) for log *a*_Na_ above −5. Such a dependence of *E* on log *a*_Na_ when log *a*_Na_ > −5 follows the Nernst equation,

*E* = const + (2.30*RT*/*F*) log *a*_Na_, (2)

where *R*, *T*, and *F* represent gas constant, absolute temperature, and Faraday constant, respectively. The value of 2.30*RT*/*F* equals 59.2 mV at 25 °C.

Figure 2b shows the E vs. log *a*_Na_ plot for the same electrode in the presence of 0.050 mol/L KCl. In the region where log *a*_Na_ is small, the *E* value is larger than when KCl is not added, and it is almost a constant value independent of log *a*_Na_. When log *a*_Na_ exceeds −4, the *E* value increases, and the Nernstian response is observed when log *a*_Na_ exceeds −3. The same trend was seen in the presence of other metal chloride salts, but the log *a*_Na_ value at which *E* begins to depend on log *a*_Na_ was different. Similar results were obtained for the electrodes with other ionophores. The *E* vs. log *a*_Na_ data for all the systems are shown in Supplementary Materials.

**Figure 3:**
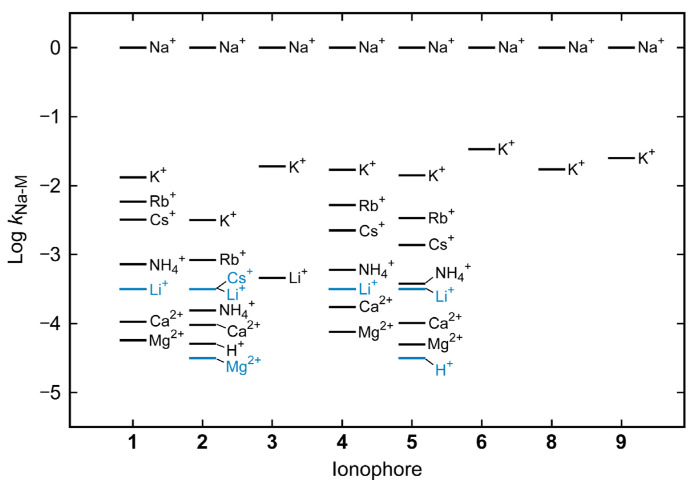
Graphical display of selectivity coefficient data in Table 1. The blue lines indicate upper limit values.

For solutions containing an interfering ion (M*^n^*^+^), the dependence of *E* on log *a*_Na_ is expressed by the following Nikolsky–Eisenman equation:

*E* = const + (2.30*RT*/*F*) log (*a*_Na_ + *k*_Na-M_ *a*_M_^1/*n*^), (3)

where *a*_M_ denotes the activity of M*^n^*^+^ and *k*_Na-M_ is the selectivity coefficient for M*^n^*^+^ with respect to Na^+^. The *k*_Na-M_ values were determined for electrodes containing the ionophores **1**–**6**, **8**, and **9** with various M^n+^ ions by the fixed interference method [15] using a nonlinear least-square fitting method with KaleidaGraph^TM^ (Synergy Software, version 3.52) based on Equation (3). The values obtained are summarized in Table 1, together with the standard errors estimated by the least-squares method. For the *k*_Na-K_ value of **2**, the mean and standard deviation of 11 measurements during the experimental period are shown because of the large change over time (see below). In addition, since the log *a*_Na_ dependence of *E* without any interfering ion follows the Nernst equation in the range of log *a*_Na_ > −5 (Figure 2a), the lower limit of the log *k*_Na-M_ value obtained under the conditions of this study is about −3.5 for Li^+^, K^+^, Rb^+^, and Cs^+^ (coexisting concentration 0.050 mol/L) and about −4.5 for Mg^2+^, Ca^2+^, NH_4_^+^, and H^+^ (coexisting concentration 0.47–0.50 mol/L). To make it easier to see the differences in selectivity, the logarithmic values of *k*_Na-M_ for each ionophore are shown on the vertical axis in Figure 3.

Although the *k*_Na-M_ values of **1** and **2** determined in this study are generally close to the literature values [11,13]; however, for *k*_Na-Li_ of **1** (log *k*_Na-Li_ = −3.0 [13]), *k*_Na-Cs_ of **1** (log *k*_Na-Cs_ = −2.0 [13]), *k*_Na-Li_ of **2** (log *k*_Na-Li_ = −2.98 [11]), and *k*_Na-H_ of **2** (log *k*_Na-H_ = −3.10 [11]), the values in this study are less than 1/3 of the literature values. These differences in results may be due to differences in the composition of the electrode membranes and analysis methods.

The *k*_Na-M_ values obtained were all smaller than 1 (log *k*_Na-M_ < 0), meaning that all the electrodes respond to Na^+^ more selectively than to the interfering ions. As can be seen from the *k*_Na-M_ values for the electrodes of **4** and **5**, these silicon-bridged bis(12-crown-4)s have the same selectivity sequence, Na^+^ > K^+^ > Rb^+^ > Cs^+^ > NH_4_^+^ > Li^+^ > Ca^2+^ > Mg^2+^ > H^+^, although the order of Li^+^ may be further back because the *k*_Na-Li_ values obtained are upper bound. This selectivity sequence is also identical to that for the malonate-bridged bis(12-crown-4) **1**, but different in part from that for the calix[4]arene derivative **2** (Na^+^ > K^+^ > Rb^+^ > Cs^+^ ~ Li^+^ > NH_4_^+^ > Ca^2+^ > H^+^ > Mg^2+^).

The absolute value of log *k*_Na-M_ shows how high the Na^+^ selectivity against M*^n^*^+^. The selectivity of the silicon-bridged bis(12-crown-4) clearly depends on the structures of hydrocarbon substituents (R_1_ and R_2_) on the silicon atom. However, the *k*_Na-K_ values for **3** (R_1_ = R_2_ = butyl), **4** (R_1_ = R_2_ = octyl), and **8** (R_1_ = methyl, R_2_ = dodecyl) are close to each other, indicating that the length and symmetry of the alkyl chains have little or no effect on the magnitude of selectivity. This suggests that the length of linear alkyl groups has little effect on the conformation and electronic state of the two crown rings.

The ionophore **5** (R_1_ = R_2_ = 2-ethylhexyl) has clearly higher Na^+^ selectivity for almost all interfering cations than the structural isomer **4** (R_1_ = R_2_ = octyl), showing that branching of the alkyl chains is effective in improving selectivity. It is noteworthy that the Na^+^ selectivity of **5** is equal to or greater than the commercial ionophore **1**; in particular, the selectivity of **5** for Na^+^ over Rb^+^, Cs^+^, and NH_4_^+^ is significantly higher. When compared to another commercial ionophore **2**, the Na^+^ selectivity of **5** is higher against H^+^ and possibly Li^+^, but lower against other ions. On the other hand, **6** (R_1_ = R_2_ = phenyl) and **9** (R_1_ = methyl, R_2_ = 2-phenylethyl), which have two or one benzene ring within the hydrocarbon substituent, have smaller selectivity for Na^+^ over K^+^ than other silicon-bridged bis(12-crown-4)s.

**Figure 4:**
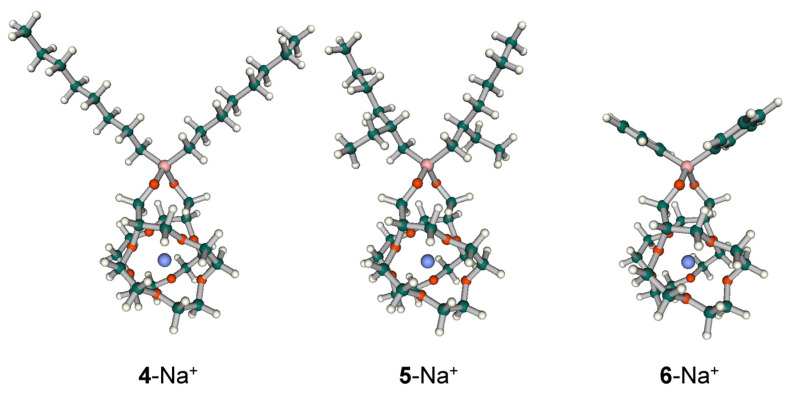
Equilibrium geometries of Na+ complexes of the ionophores (SS type) 4, 5, and 6 obtained by DFT calculations. All of the atoms are colored by the type of element (white: H, green: C, red: O, blue: Na, pink: Si). The structures are drawn by Facio software (version 23.1.5) [22].

To discuss the difference in Na^+^-K^+^ selectivity of **4**, **5**, and **6**, the equilibrium geometries in vacuum of these Na^+^ and K^+^ complexes were calculated by using a hybrid DFT method (B3LYP [16,17,18]) with the Gaussian16 program package [19]. In this calculation, the 6-31G(d) standard basis set was used for H, C, and O, and the LANL2DZ effective core potential [20] for Na, K, and Si. The charge distribution was evaluated by natural bond orbital analysis [21]. Each of the ionophores is strictly a mixture of RR, RS, and SS isomers because the two 12-crown-4 moieties in the molecule each have one chiral carbon atom; DFT calculations were performed only for the SS-isomer. The results are summarized in Supplementary Materials. As an example, the equilibrium geometries of Na^+^ complexes of these ionophores are shown in Figure 4. The ionophores have a sandwich structure in which one alkali metal ion is held by two 12-crown-4 rings.

Interestingly, there was no difference between the Na^+^ complexes of the three ionophores with respect to the Na^+^–O distance (average 0.252 nm) and the natural charges of O (average −0.62) and Na^+^ (0.87). It was the same for the K^+^ complexes, with a K^+^–O distance of 0.282 nm (average) and natural charges of 0.61 (average) and 0.91 for O and K^+^, respectively, for all the ionophores. Furthermore, the differences in the thermochemical quantities between the Na^+^ and K^+^ complexes of the same ionophore, which were obtained from frequency calculations of the Gaussian program, were also almost identical for the three ionophores. These results suggest that differences in hydrocarbon side chains have little effect on the binding conformation and energy between the alkali metal ion and the crown ether moieties in the most stable geometry of the complex. Thus, conformational freedom of the crown ether moieties may govern the differences in selectivity of these ionophores. Figure 4 shows that the steric crowding around the silicon atoms by the two hydrocarbon side chains appears to increase in the order **6**, **4**, and **5**. In fact, reflecting this, the O–Si–O bond angles in the Na^+^ and K^+^ complexes of these ionophores are smaller in the order of **6** (Na^+^ complex: 110.24°, K^+^ complex: 110.74°), **4** (Na^+^ complex: 108.10°, K^+^ complex: 108.48°), and **5** (Na^+^ complex: 107.23°, K^+^ complex: 107.56°). One can speculate that steric crowding around the silicon atoms prevents the two crown ether moieties from forming a conformation away from each other, making it easier to take a conformation favorable to sandwiching the smaller Na^+^ ion (note that Li^+^ is not sandwiched in size with 12-crown-4). The same should be true for the RR and RS isomers.

### 2.3. Stability of Ion-Selective Electrodes

**Figure 5:**
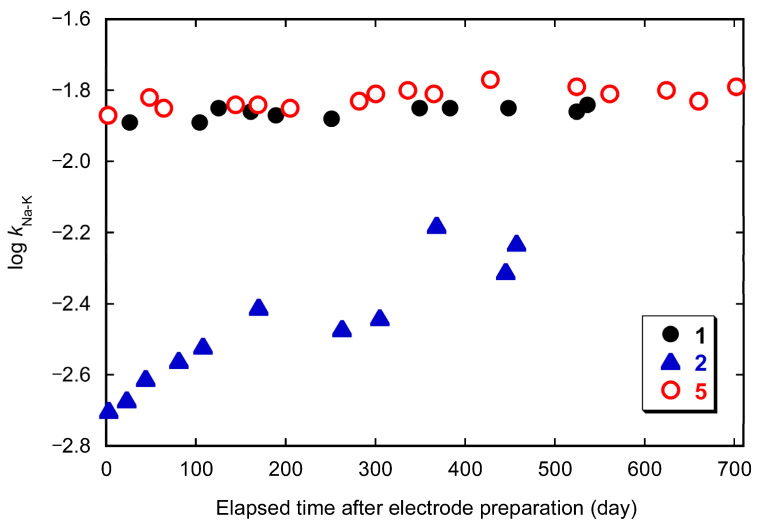
Dependence of Na^+^-K^+^ selectivity coefficient (*k*_Na-K_) on elapsed time after electrode preparation for the electrodes of ionophores **1**, **2**, and **5**.

The *k*_Na-K_ values of the electrodes with **1**, **2**, and **5** were repeatedly measured over a long period of time to evaluate the stability of the electrodes. During the interval period between measurements, the electrode cap with the membrane was removed from the electrode and stored in a sealed container after air-drying. Figure 5 shows the dependence of log *k*_Na-K_ values on the number of days elapsed after electrode fabrication. The act of immersing the electrode in the sample solution and measuring the potential, performed on each measurement day, averaged about 50 times. Thus, Figure 4 shows that the Na^+^-K^+^ selectivity of electrodes **5** remained almost constant over 800 measurements for about 2 years. There was concern that the silane compound would be susceptible to hydrolysis, but the electrode of **5** is as stable as or more stable than the electrode of **1**. This may indicate that the large alkyl group of **5** makes it sufficiently hydrophobic and less susceptible to hydrolysis. On the other hand, the *k*_Na-K_ values of the electrode with the calix[4]arene derivative **2** increase with time immediately after electrode preparation, which indicates a marked decrease in Na^+^-K^+^ selectivity. Thus, the electrode with **2** is significantly inferior to the electrodes with **1** and **5** in stability.

## 3. Materials and Methods

### 3.1. Reagents and Instruments

2-Hydroxymethyl-12-crown-4 (12C4-CH_2_OH) (Sigma-Aldrich/Merck, Darmstadt, Germany, 95.0%) was dried with Molecular Sieves 3A 1/16 for more than 24 h. Dibutyldichlorosilane (TCI, Tokyo, Japan, >94.0%), dichlorodioctylsilane (Sigma-Aldrich/Merck, >98.0%), dichlorobis(2-ethylhexyl)silane (TCI, >95.0%), dichlorodiphenylsilane (TCI, >98.0%), dichloromethyloctylsilane (TCI, >97.0%), dichlorododecylmethylsilane (TCI, >95.0%), and dichloromethyl(2-phenylethyl)silane (TCI, >98.0%) were purchased and used without purification. Bis[(12-crown-4)methyl]-2-dodecyl-2-methylmalonate **1** (Dojindo Laboratories, Kumamoto, Japan, >95.0%), tetraethyl 4-*tert*-butylcalix[4]arene-*O*,*O*′,*O*″,*O*‴-tetraacetate **2** (Sigma-Aldrich/Merck, >97%), *o*-nitrophenyl octyl ether (Dojindo Laboratories, >99.0%), sodium tetrakis [3,5-bis(trifluoromethyl)phenyl]borate (Dojindo Laboratories, >99.0%), and tetrahydrofuran (Wako Pure Chemical Industries, Osaka, Japan, for Spectrochemical Analysis) were also used as received. Methanol (Kanto Chemical, Tokyo, Japan, for LC/MS) was distilled. PVC (Wako Pure Chemical Industries) was dissolved in tetrahydrofuran, then precipitated by adding distilled methanol and filtered; after repeating this operation three times, the precipitate was dried under reduced pressure. Water was deionized with a Kurita Demi-Ace Model DX-15A demineralizer and further purified with a Millipore Simplicity UV System. All other chemicals were of guaranteed reagent grade and were used without further purification.

Identification of the synthetic products was carried out using a JNM-ECS400 FT-NMR spectrometer (JEOL, Tokyo, Japan) and a Exactive mass spectrometer (Thermo Fisher Scientific, Waltham, MS, USA) at the Center for Analytical Instrumentation, Chiba University. GC-FID analysis of the products was performed on a GC-14B gas chromatograph (Shimadzu, Kyoto, Japan) equipped with a CBP1-M25-025 fused silica capillary column (length: 25 m, I.D.: 0.22 mm, film thickness: 0.25 μm) with the injector, column, and detector temperatures of 320, 300, and 320 °C, respectively. A F-72 pH/ion meter (Horiba, Kyoto, Japan) was used as a potentiometer for performance testing of ion-selective electrodes along with a 2565A double-junction type reference electrode.

### 3.2. Synthesis

#### 3.2.1. Bis[(12-crown-4)methoxy]dibutylsilane **3**

0.767 g (0.00372 mol) of 12C4-CH_2_OH and 0.381 g (0.00179 mol) of dibutyldichlorosilane were mixed and reacted at room temperature under a dry nitrogen atmosphere with continuous stirring. The endpoint of the reaction was after 30 h, at which time gaseous HCl was no longer detected with the pH test paper. To separate the target product from a small excess of raw material (12C4-CH_2_OH) and byproduct HCl, 10 mL of toluene and 10 mL of deionized water were added to the resulting reaction solution and shaken for 5 min. The toluene phase was further washed twice with 10 mL of deionized water, and the aqueous phase was confirmed to be neutral with pH test paper. The toluene phase was taken out, the solvent was removed, and the residue was dried under reduced pressure over diphosphorus pentoxide. Yield: 0.813 g (0.00147 mol, 82.2%). ^1^H NMR (400 MHz, CDCl_3_) *δ* (ppm): 0.61 (t, *J* = 6.6 Hz, 4H, CH_2_ of butyl), 0.88 (t, *J* = 7.0 Hz, 6H, CH_3_ of butyl), 1.31−1.35 (m, 8H, CH_2_ of butyl), 3.62−3.76 (m, 34H, (12-crown-4)methoxy). HR MS (ESI+) *m*/*z*: calcd for C_26_H_56_NO_10_Si^+^ [M + NH_4_]^+^ 570.3674, found 570.3668; calcd for C_26_H_52_O_10_SiNa^+^ [M + Na]^+^ 575.3227, found 575.3221; calcd for C_26_H_52_O_10_SiK^+^ [M + K]^+^ 591.2967, found 591.2958.

#### 3.2.2. Bis[(12-crown-4)methoxy]dioctylsilane **4**

The product was obtained from 0.904 g (0.00438 mol) of 12C4-CH_2_OH and 0.691 g (0.00212 mol) of dichlorodioctylsilane in a similar manner to the synthesis of **3**. The reaction time was about 40 h. Yield: 1.243 g (0.00187 mol, 88.2%). ^1^H NMR (400 MHz, CDCl_3_) *δ* (ppm): 0.60 (t, *J* = 8.2 Hz, 4H, CH_2_ of octyl), 0.88 (t, *J* = 7.0 Hz, 6H, CH_3_ of butyl), 1.26−1.43 (m, 24H, CH_2_ of octyl), 3.62−3.76 (m, 34H, CH and CH_2_ of (12-crown-4)methoxy). HR MS (ESI+) m/z: calcd for C_34_H_72_NO_10_Si^+^ [M + NH_4_]^+^ 682.4926, found 682.4932; calcd for C_34_H_68_O_10_SiNa^+^ [M + Na]^+^ 687.4479, found 687.4467; calcd for C_34_H_68_O_10_SiK^+^ [M + K]^+^ 703.4219, found 703.4200.

#### 3.2.3. Bis[(12-crown-4)methoxy]bis(2-ethylhexyl)silane **5**

The product was obtained from 0.954 g (0.00463 mol) of 12C4-CH_2_OH and 0.734 g (0.00225 mol) of dichlorobis(2-ethylhexyl)silane in a similar manner to the synthesis of **3**. The reaction time was about 50 h. Yield: 1.294 g (0.00195 mol, 86.3%). ^1^H NMR (400 MHz, CDCl_3_) *δ* (ppm): 0.60 (d, *J* = 6.4 Hz, 4H, CH2 of hexyl), 0.83 (t, *J* = 7.6 Hz, 6H, CH_3_ of hexyl), 0.89 (t, *J* = 7.0 Hz, 6H, CH_3_ of ethyl), 1.19−1.37 (m, 14H, CH and CH_2_ of hexyl), 1.46−1.49 (m, 4H, CH_2_ of ethyl), 3.45−3.87 (m, 34H, CH and CH_2_ of (12-crown-4)methoxy). HR MS (ESI+) m/z: calcd for C_34_H_72_NO_10_Si^+^ [M + NH_4_]^+^ 682.4926, found 682.4913; calcd for C_34_H_68_O_10_SiNa^+^ [M + Na]^+^ 687.4479, found 687.4465; calcd for C_34_H_68_O_10_SiK^+^ [M + K]^+^ 703.4219, found 703.4202.

#### 3.2.4. Bis[(12-crown-4)methoxy]diphenylsilane **6**

The product was obtained from 0.845 g (0.00410 mol) of 12C4-CH_2_OH and 0.427 g (0.00169 mol) of dichlorodiphenylsilane in a similar manner to the synthesis of **3**. The reaction time was about 25 h. Yield: 0.889 g (0.00150 mol, 88.7%). ^1^H NMR (400 MHz, CDCl_3_) *δ* (ppm): 3.60–3.78 (m, 34H, CH and CH_2_ of (12-crown-4)methoxy), 7.35–7.43 (m, 6H, CH of phenyl), 7.63–7.66 (m, 4H, CH of phenyl). HR MS (ESI+) m/z: calcd for C_30_H_48_NO_10_Si^+^ [M + NH_4_]^+^ 610.3048 found 610.3045; calcd for C_30_H_44_O_10_SiNa^+^ [M + Na]^+^ 615.2601, found 615.2592; calcd for C_30_H_44_O_10_SiK^+^ [M + K]^+^ 631.2341, found 631.2323.

#### 3.2.5. Bis[(12-crown-4)methoxy]methyloctylsilane **7**

The product was obtained from 0.767 g (0.00372 mol) of 12C4-CH_2_OH and 0.381 g (0.00168 mol) of dichloromethyloctylsilane in a similar manner to the synthesis of **3**. The reaction time was about 24 h. Yield: 0.832 g (0.00147 mol, 87.4%). 1H NMR (400 MHz, CDCl_3_) *δ* (ppm): 0.10 (t, *J* = 2.0 Hz, 3H, CH_3_ of methyl), 0.60 (t, *J* = 8.2 Hz, 2H, CH_2_ of octyl), 0.88 (t, *J* = 7.0 Hz, 3H, CH_3_ of octyl), 1.26–1.36 (m, 12H, CH_2_ of octyl), 3.45–3.90 (m, 34H, CH and CH_2_ of (12-crown-4)methoxy). HR MS (ESI+) m/z: calcd for C_27_H_58_NO_10_Si^+^ [M + NH_4_]^+^ 584.3830 found 584.3820; calcd for C_27_H_54_O_10_SiNa^+^ [M + Na]^+^ 589.3384, found 589.3368; calcd for C_27_H_54_O_10_SiK^+^ [M + K]^+^ 605.3123, found 605.3109.

#### 3.2.6. Bis[(12-crown-4)methoxy]dodecylmethylsilane **8**

The product was obtained from 0.992 g (0.00410 mol) of 12C4-CH_2_OH and 0.650 g (0.00229 mol) of dichlorododecylmethylsilane in a similar manner to the synthesis of **3**. The reaction time was about 25 h. Yield: 1.25 g (0.00200 mol, 87.3%). ^1^H NMR (400 MHz, CDCl_3_) *δ* (ppm): 0.10 (s, 3H, CH_3_ of methyl), 0.60 (t, *J* = 8.0 Hz, 2H, CH_2_ of dodecyl), 0.88 (t, *J* = 6.8 Hz, 3H, CH_3_ of dodecyl), 1.25–1.30 (m, 20H, CH_2_ of dodecyl), 3.46–3.86 (m, 34H, CH and CH_2_ of (12-crown-4)methoxy). HR MS (ESI+) *m*/*z*: calcd for C_31_H_66_NO_10_Si^+^ [M + NH_4_]^+^ 640.4456, found 640.4445; calcd for C_31_H_62_O_10_SiNa^+^ [M + Na]^+^ 645.4010, found 645.3996; calcd for C_31_H_62_O_10_SiK^+^ [M + K]^+^ 661.3749, found 661.3733.

#### 3.2.7. Bis[(12-crown-4)methoxy]methyl(2-phenylethyl)silane **9**

The product was obtained from 0.641 g (0.00311 mol) of 12C4-CH_2_OH and 0.323 g (0.00147 mol) of dichloromethyl(2-phenylethyl)silane in a similar manner to the synthesis of **3**. The reaction time was about 22 h. Yield: 0.654 g (0.00195 mol, 79.4%). ^1^H NMR (400 MHz, CDCl_3_) *δ* (ppm): 0.12 (s, 3H, CH_3_), 0.98 (t, *J* = 9.4 Hz, 2H, CH_2_ of ethyl), 2.69 (t, *J* = 9.0 Hz, 2H, CH_2_ of ethyl), 3.63–3.75 (m, 34H, CH and CH_2_ of (12-crown-4)methoxy), 7.15–7.29 (m, 5H, CH of phenyl). HR MS (ESI+) *m*/*z*: calcd for C_27_H_50_NO_10_Si^+^ [M + NH_4_]^+^ 576.3204, found 576.3188; calcd for C_27_H_46_O_10_SiNa^+^ [M + Na]^+^ 581.2758, found 581.2744; calcd for C_27_H_46_O_10_SiK^+^ [M + K]^+^ 597.2497, found 597.2482.

### 3.3. Electrode Preparation and Potentiometric Measurement Experiments

A mixture of PVC (23–31%), *o*-nitrophenyl octyl ether (65–72%), ionophore (3–5%), and sodium tetrakis [3,5-bis(trifluoromethyl)phenyl]borate (0.6–1%) was dissolved in tetrahydrofuran (3 mL). One drop of the solution was dropped onto a PTFE membrane filter (Sumitomo Electric Industries FP-100, pore diameter 1 μm, thickness 0.1 mm, diameter 5 mm) attached to an electrode cap (DKK-TOA 4031) and air-dried at room temperature for 10 min; after this process was repeated 10 times, the membrane was further air-dried overnight. The electrode cap was incorporated into an Ag-AgCl electrode (DKK-TOA 7904L). An aqueous solution of 1.0 mol/L NaCl was injected into the electrode as an internal solution, and the electrode was immersed in the NaCl solution for 24 h for conditioning. The electrode was attached to a pH/ion meter together with a double-junction type Ag-AgCl reference electrode, which was injected with aqueous solutions of 3.3 mol/L KCl and 0.10 mol/L NH_4_NO_3_ as inner and outer chamber solutions, respectively. The electrochemical cell system when the electrodes were immersed in a test solution was expressed as follows: Ag, AgCl/3.3 mol/L KCl/0.10 mol/L NH_4_NO_3_//test solution/polymer membrane/1.0 mol/L NaCl/AgCl, Ag. The test solutions are aqueous solution containing only NaCl (1.0 × 10^−6^−1.0 × 10^−1^ mol/L) or both NaCl (1.0 × 10^−6^−1.0 × 10^−1^ mol/L) and a chloride salt of an interfering cation (0.050 mol/L LiCl, KCl, RbCl, and CsCl; 0.47–0.50 mol/L MgCl_2_, CaCl_2_, NH_4_Cl, and HCl), which were placed in a thermostatic bath at 25 ± 0.1 °C and stirred with a magnetic stirrer during the emf measurement. The measured potential generally stabilized within 1 min, but to be sure, the potential was read after 5 min. The measurement was repeated three times for one test solution and the average value was obtained. After one day of measurements, the electrode cap with the membrane was removed from the electrode, rinsed with deionized water, air-dried at room temperature, and stored in a sealed container until the next measurement day.

## 4. Conclusions

We have successfully synthesized silicon-bridged bis(12-crown-4)s with a structure similar to that of malonate-bridged bis(12-crown-4)(1), one of the conventional Na^+^ ionophores. These compounds can be easily synthesized by a one-step reaction at room temperature in a solvent-free manner using commercially available raw materials. Conventional Na^+^ ionophores are expensive, and their applications have been limited to ultra-trace scale, such as sensors, but the new compounds are easy to synthesize and inexpensive, so they are expected to be used in applications requiring macro quantities, such as extractants for Na^+^.

The performance of PVC membrane-type ion-selective electrodes utilizing these silicon-bridged bis(12-crown-4)s was evaluated. The results showed that the electrodes exhibit high Na^+^ selectivity and that the selectivity depends on the structures of hydrocarbon substituents on the silicon atoms. The compound with two 2-ethylhexyl groups (5) has particularly excellent Na^+^ selectivity, and the performance of the electrode was found to be equal to or better than that of the previously used electrode with bis[(12-crown-4)methyl]-2-dodecyl-2-methylmalonate (1). Even compared to an electrode using tetraethyl 4-*tert*-butylcalix[4]arene-*O*,*O*′,*O*″,*O*‴-tetraacetate (2), which is known for its very high Na^+^ selectivity, the electrode of 5 has higher Na^+^ selectivity in the presence of Li^+^ and H^+^ and is more stable over time. The silicon-bridged bis(12-crown-4)s synthesized in this study are expected to have practical applications due to their balance of high Na^+^ selectivity and stability.

## 5. Patents

A patent application related to this research has been filed (Japan Patent JP2024-125964A).

## Figures and Tables

**Table 1:** Selectivity coefficients for interfering cations with respect to Na^+^ at 25 °C *.

Ionophore		Log *k*_Na-M_ ^†^							
	M =	Li^+^	K^+^	Rb^+^	Cs^+^	Mg^2+^	Ca^2+^	NH_4_^+^	H^+^
**1**		<−3.5	−1.88 (0.01)	−2.23 (0.02)	−2.49 (0.02)	−4.24 (0.07)	−3.97 (0.01)	−3.14 (0.01)	
**2**		<−3.5	−2.5 ± 0.2 ^†^	−3.08 (0.05)	<−3.5	<−4.5	−4.02 (0.01)	−3.81 (0.03)	−4.29 (0.01)
**3**		−3.34 (0.09)	−1.72 (0.03)						
**4**		<−3.5	−1.77 (0.01)	−2.28 (0.01)	−2.65 (0.03)	−4.12 (0.06)	−3.76 (0.02	−3.22 (0.02)	
**5**		<−3.5	−1.85 (0.01)	−2.47 (0.02)	−2.86 (0.02)	−4.30 (0.04)	−3.99 (0.01)	−3.42 (0.01)	<−4.5
**6**			−1.47 (0.05)				-		
**8**			−1.76 (0.01)						
**9**			−1.60 (0.04)						

* Values in parentheses represent standard errors estimated by the nonlinear least squares method. ^†^ Mean and standard deviation of 11 measurements.

## Data Availability

The original contributions presented in this study are included in the article/Supplementary Materials. Further inquiries can be directed to the corresponding author.

## Supplementary Materials

The following supporting information can be downloaded at https://www.mdpi.com/article/10.3390/molecules30040925/s1, Figure S1: ^1^H NMR spectrum of **3**; Figure S2: Mass spectrum of **3**; Figure S3: ^1^H NMR spectrum of **4**; Figure S4: Mass spectrum of **4**; Figure S5: ^1^H NMR spectrum of **5**; Figure S6: Mass spectrum of **5**; Figure S7: ^1^H NMR spectrum of **6**; Figure S8: Mass spectrum of **6**; Figure S9: ^1^H NMR spectrum of **7**; Figure S10: Mass spectrum of **7**; Figure S11: ^1^H NMR spectrum of **8**; Figure S12: Mass spectrum of **8**; Figure S13: ^1^H NMR spectrum of **9**; Figure S14: Mass spectrum of **9**; Table S1: Emf (*E*) data of ion-selective electrodes measured at 25 °C; Table S2: Summary of the results of DFT calculations.

## References

[1] Eijkelkamp N., Linley J.E., Baker M.D., Minett M.S., Cregg R., Werdehausen R., Rugiero F., Wood J.N. (2012). Neurological perspectives on voltage-gated sodium channels. Brain.

[2] Bernal A., Zafra M.A., Simón M.J., Mahía J. (2023). Sodium homeostasis, a balance necessary for life. Nutrients.

[3] World Health Organization (2003). Sodium in Drinking-Water: Background Document for Development of WHO Guidelines for Drinking-Water Quality.

[4] Ploegaerts G., Desmet C., Van Krieken M. (2016). Assay of sodium in food: Comparison of different preparation methods and assay techniques. J. Food Compost. Anal..

[5] Buzanovskii A. (2018). Determination of sodium in blood. Rev. J. Chem..

[6] Khan N.J., Tariq R., Saleem H., Mushtaq M.W. (2023). Quantification of sodium from food sources by using various analytical techniques. Biologia.

[7] Nielsen S.S., Ismail B.P., Nielsen S.S. (2024). Sodium determination using ion-selective electrodes, mohr titration, and test strips. Nielsen’s Food Analysis Laboratory Manual.

[8] Suzuki K., Sato K., Hisamoto H., Siswanta D., Hayashi K., Kasahara N., Watanabe K., Yamamoto N., Sakakura H. (1996). Design and synthesis of sodium ion-selective ionophores based on 16-crown-5 derivatives for an ion-selective electrode. Anal. Chem..

[9] Cadogan A.M., Diamond D., Smyth M.R., Deasy M., McKervey M.A., Harris S.J. (1989). Sodium-selective polymeric membrane electrodes based on calix[4]arene ionophores. Analyst.

[10] Kimura K., Miura T., Matsuo M., Shono T. (1990). Polymeric membrane sodium-selective electrodes based on lipophilic calix[4]arene derivatives. Anal. Chem..

[11] Shibutani Y., Yoshinaga H., Yakabe K., Shono T., Tanaka M. (1994). Polymeric membrane sodium-selective electrodes based on calix[4]arene ionophores. J. Incl. Phenom. Mol. Recognit. Chem..

[12] Tamura H., Kimura K., Shono T. (1982). Coated wire sodium- and potassium-selective electrodes based on bis(crown ether) compounds. Anal. Chem..

[13] Shono T., Okahara M., Ikeda I., Kimura K., Tamura H. (1982). Sodium-selective PVC membrane electrodes based on bis(12-crown-4)s. J. Electroanal. Chem..

[14] Kielland J. (1937). Individual activity coefficients of ions in aqueous solutions. J. Am. Chem. Soc..

[15] Umezawa Y., Bühlmann P., Umezawa K., Tohda K., Amemiya S. (2000). Potentiometric selectivity coefficients of ion-selective electrodes: Part I. Inorganic cations (technical report). Pure Appl. Chem..

[16] Becke A.D. (1993). Density-functional thermochemistry. III. The role of exact exchange. J. Chem. Phys..

[17] Lee C., Yang W., Parr R.G. (1988). Development of the Colle-Salvetti correlation-energy formula into a functional of the electron density. Phys. Rev. B.

[18] Stephens P.J., Devlin F.J., Chabalowski C.F., Frisch M.J. (1994). Ab initio calculation of vibrational absorption and circular dichroism spectra using density functional force fields. J. Phys. Chem..

[19] Frisch M.J., Trucks G.W., Schlegel H.B., Scuseria G.E., Robb M.A., Cheeseman J.R., Scalmani G., Barone V., Petersson G.A., Nakatsuji H. (2016). Gaussian 16 Rev. B.01.

[20] Roy L.E., Hay P.J., Martin R.L. (2008). Revised Basis Sets for the LANL Effective Core Potentials. J. Chem. Theory Comput..

[21] Reed A.E., Curtiss L.A., Weinhold F. (1988). Intermolecular interactions from a natural bond orbital, donor-acceptor viewpoint. Chem. Rev..

[22] Suenaga M. (2008). Development of GUI for GAMESS/FMO calculation. J. Comput. Chem. Jpn..

