# Supplementary material for: Silicon-Bridged Bis(12-crown-4) Ethers as Ionophores for Sodium Ion-Selective Electrodes
Source: Molecules. 2025 Feb 17;30(4):925. doi: 10.3390/molecules30040925 (PMC11858547; doi:10.3390/molecules30040925)
Supplement: Supplementary file 1 [file molecules-30-00925-s001.zip › molecules-3429863-supplementary.pdf]

Supporting Information

Silicon-bridged bis(12-crown-4) ethers as ionophores for sodium ion-selective electrodes

Shoichi Katsuta[1]*, Yoshiyasu Ino[2], Hiroto Wakabayashi[2]

[1] Department of Chemistry, Graduate School of Science, Chiba University, 1-33 Yayoi-cho, Inage, Chiba 263-8522, Japan
[2] Department of Chemistry, Division of Advanced Science and Engineering, Graduate School of Science and Engineering, Chiba University, 1-33 Yayoi-cho, Inage, Chiba 263-8522, Japan
* Correspondence: katsuta@faculty.chiba-u.jp

Table of Contents

Figure S1. ^{1}H NMR spectrum of ionophore **3** **p.3**

Figure S2. Mass spectrum of ionophore **3** **p.4**

Figure S3. ^{1}H NMR spectrum of ionophore **4** **p.5**

Figure S4. Mass spectrum of ionophore **4** **p.6**

Figure S5. ^{1}H NMR spectrum of ionophore **5** **p.7**

Figure S6. Mass spectrum of ionophore **5** **p.8**

Figure S7. ^{1}H NMR spectrum of ionophore **6** **p.9**

Figure S8. Mass spectrum of ionophore **6** **p.10**

Figure S9. ^{1}H NMR spectrum of ionophore **7** **p.11**

Figure S10. Mass spectrum of ionophore **7** **p.12**

Figure S11. ^{1}H NMR spectrum of ionophore **8** **p.13**

Figure S12. Mass spectrum of ionophore **8** **p.14**

Figure S13. ^{1}H NMR spectrum of ionophore **9** **p.15**

Figure S14. Mass spectrum of ionophore **9** **p.16**

Table S1. Emf (*E*) data of ion-selective electrodes measured at 25 °C **p.17**

Table S2. Summary of the results of DFT calculations **p.20**

4-Na^+ complex **p.20**

4-K^+ complex **p.24**

5-Na^+ complex **p.28**

5-K^+ complex **p.32**

6-Na^+ complex **p.36**

6-K^+ complex **p.39**

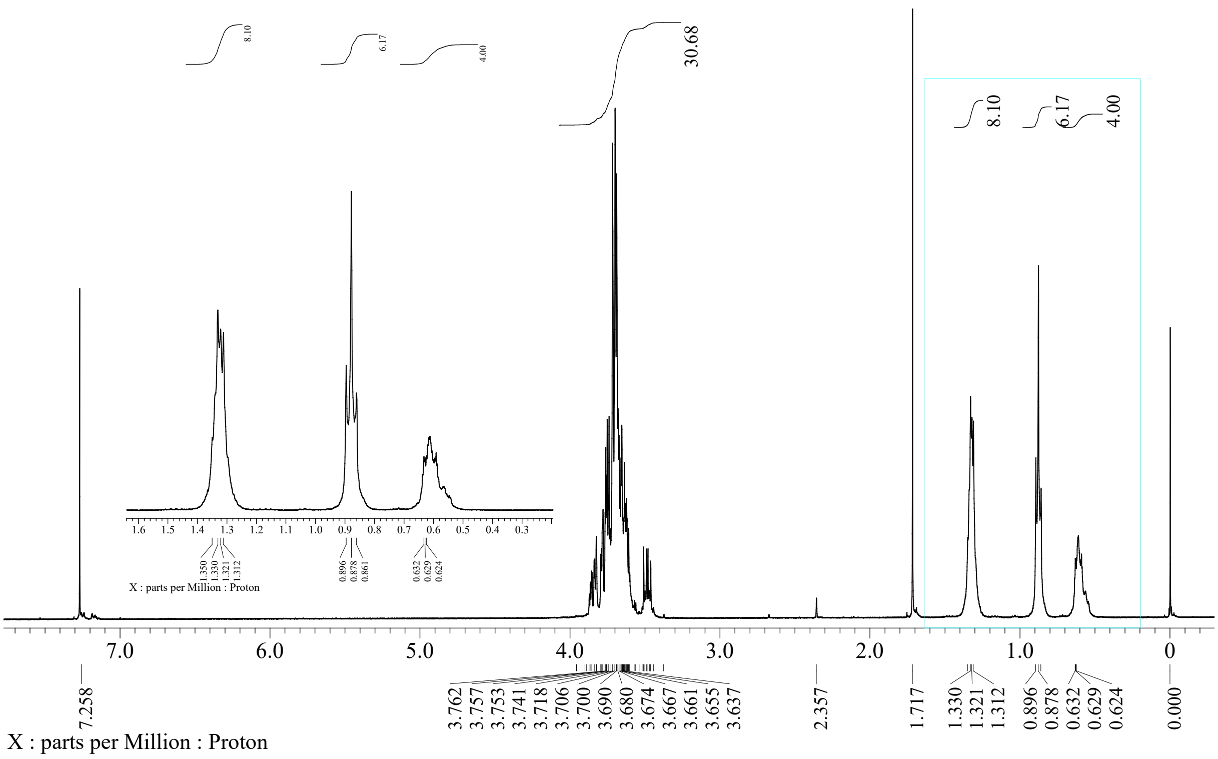

Figure S1. ^{1}H NMR spectrum of **3** (400 MHz; $CDCl_3$; Me_4Si).

The peaks at 1.72 ppm and 7.26 ppm are assigned to protons of H_2O and $CHCl_3$, respectively.

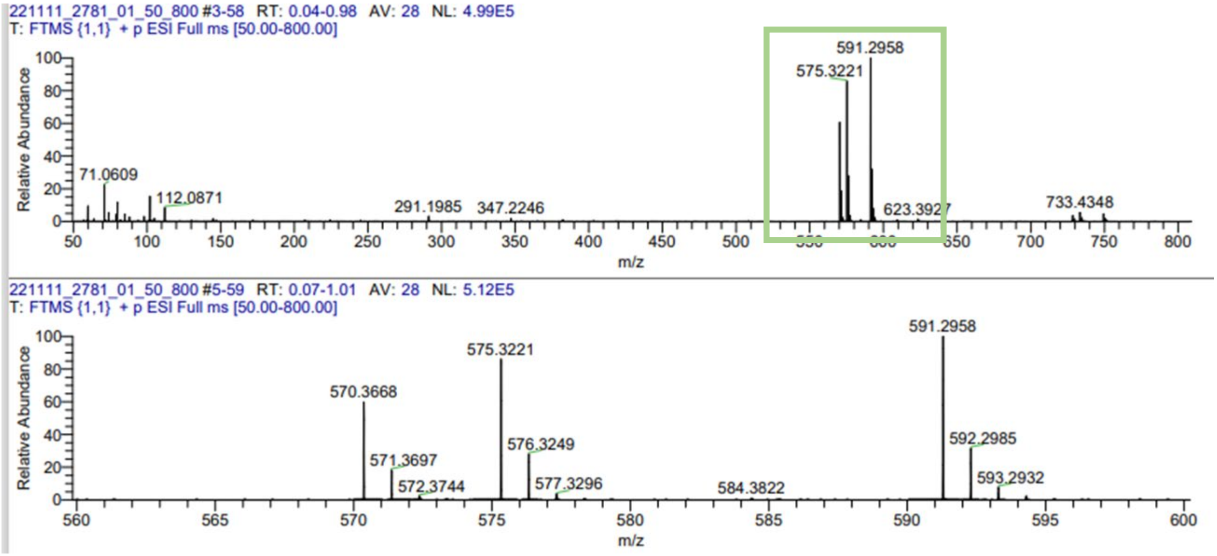

Figure S2. Mass spectrum of **3** (ESI+; CH_3CN).

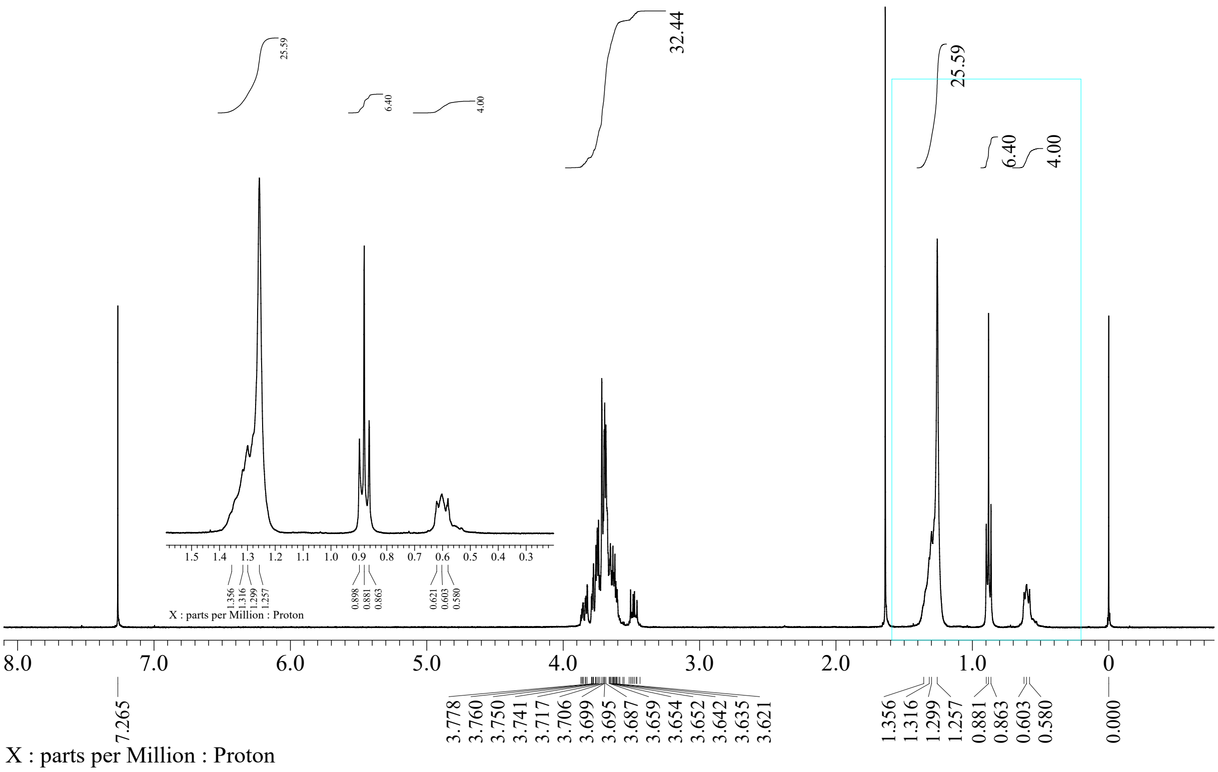

Figure S3. ^{1}H NMR spectrum of **4** (400 MHz; $CDCl_3$; Me_4Si).

The peaks at 1.63 ppm and 7.26 ppm are assigned to protons of H_2O and $CHCl_3$, respectively.

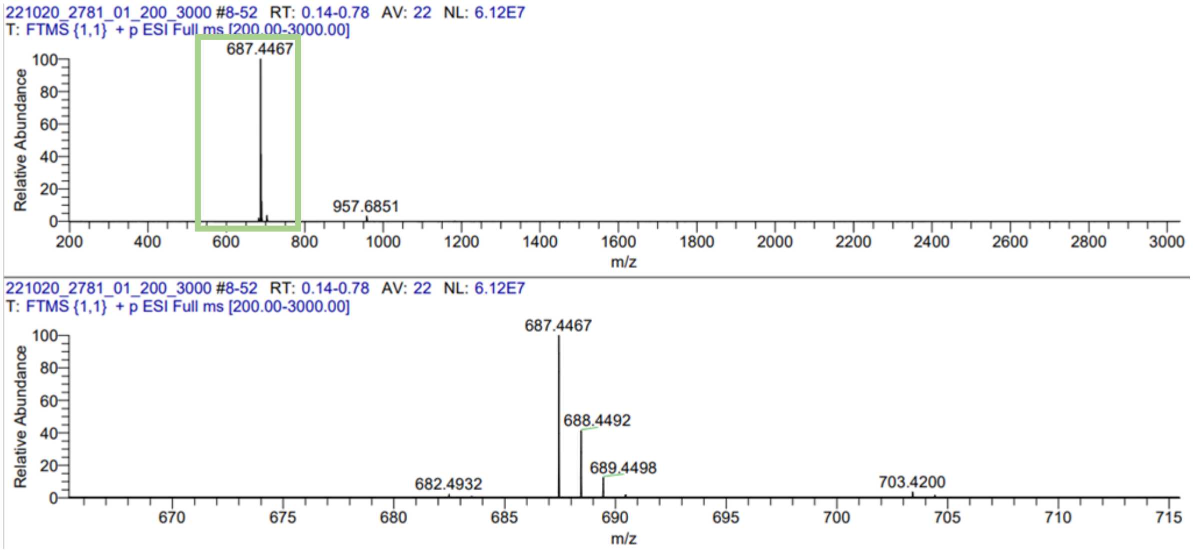

Figure S4. Mass spectrum of **4** (ESI+; CH_3CN).

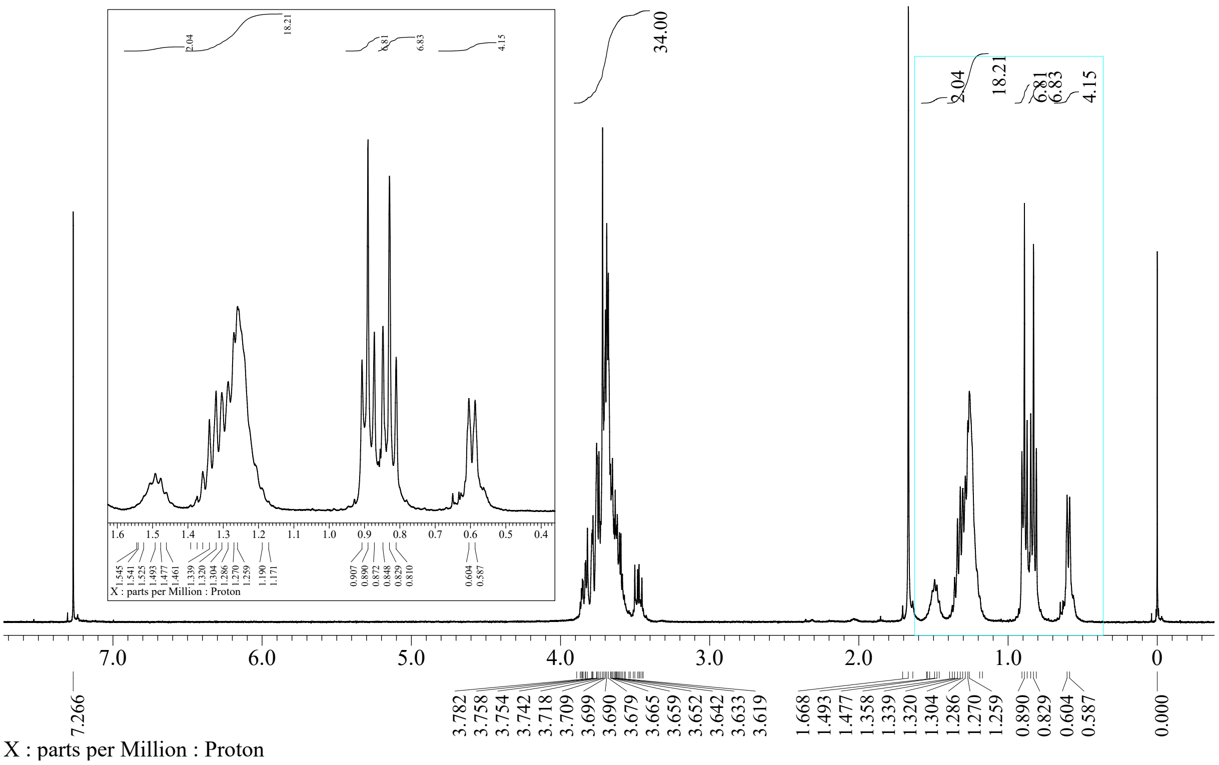

Figure S5. ^{1}H NMR spectrum of **5** (400 MHz; $CDCl_3$; Me_4Si).

The peaks at 1.68 ppm and 7.27 ppm are assigned to protons of H_2O and $CHCl_3$, respectively.

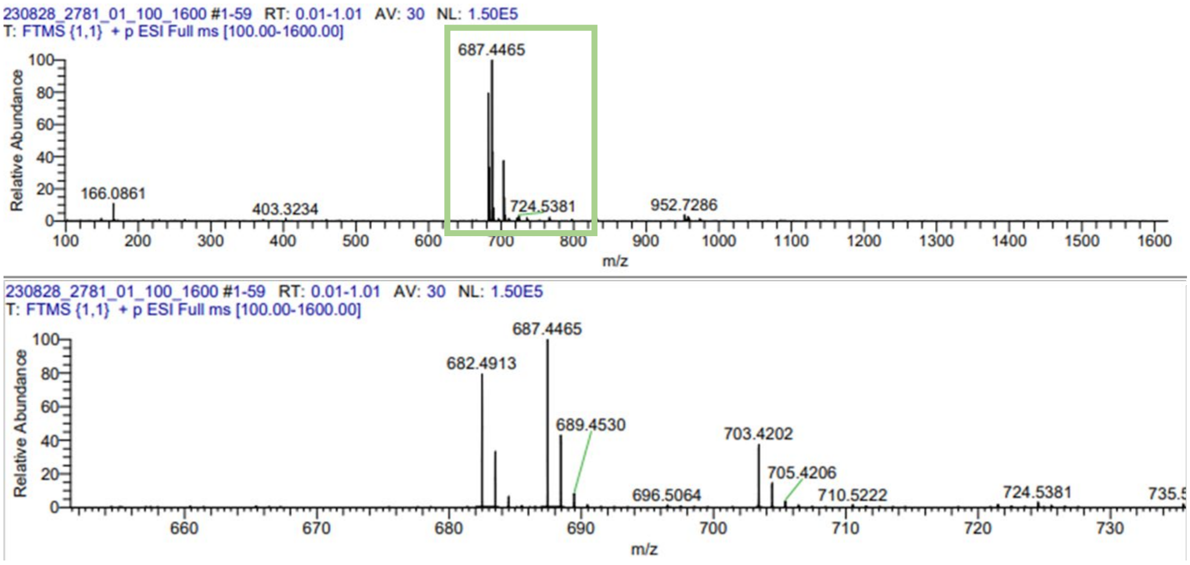

Figure S6. Mass spectrum of **5** (ESI+; CH_3CN).

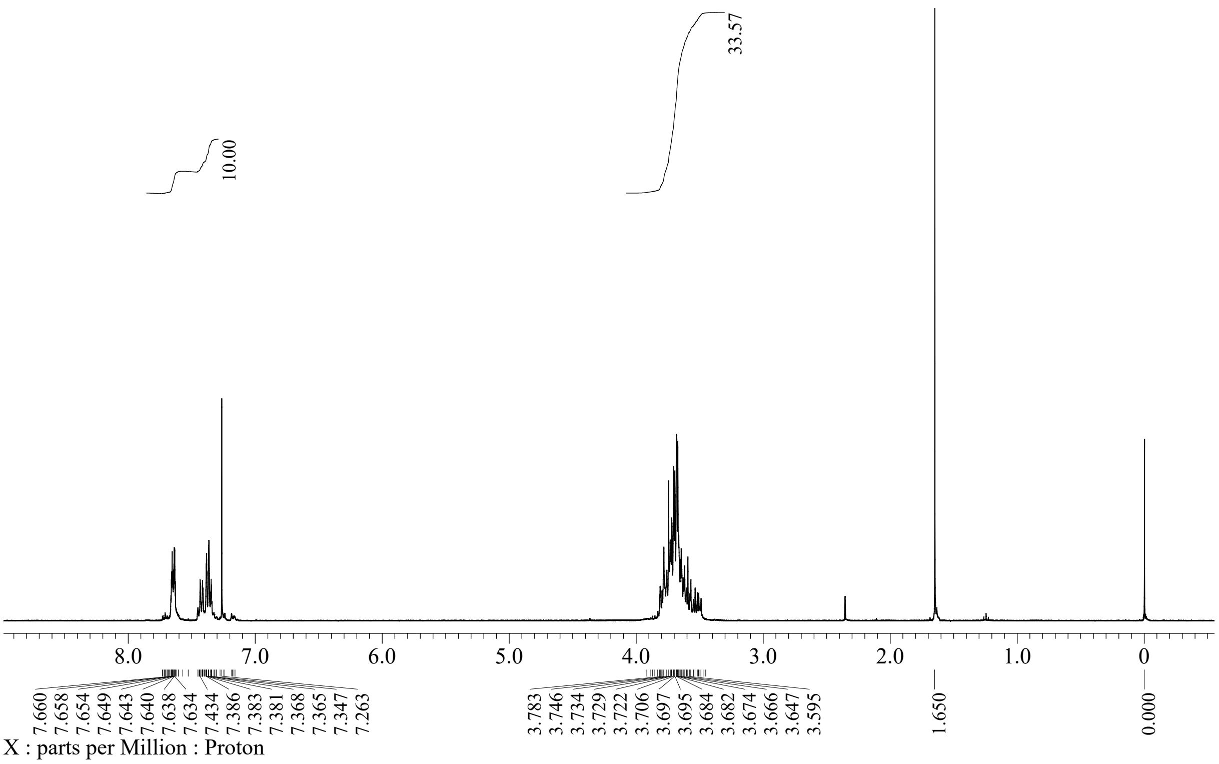

Figure S7. ^{1}H NMR spectrum of **6** (400 MHz; $CDCl_3$; Me_4Si).

The peaks at 1.65 ppm and 7.26 ppm are assigned to protons of H_2O and $CHCl_3$, respectively.

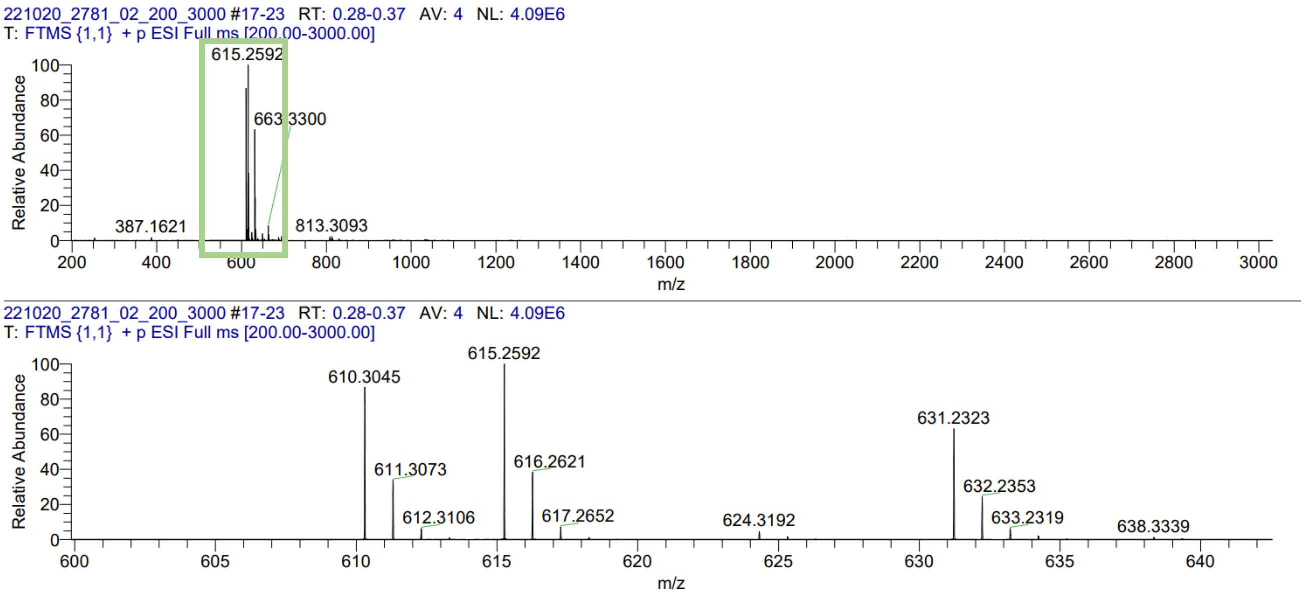

Figure S8. Mass spectrum of **6** (ESI+; CH_3CN).

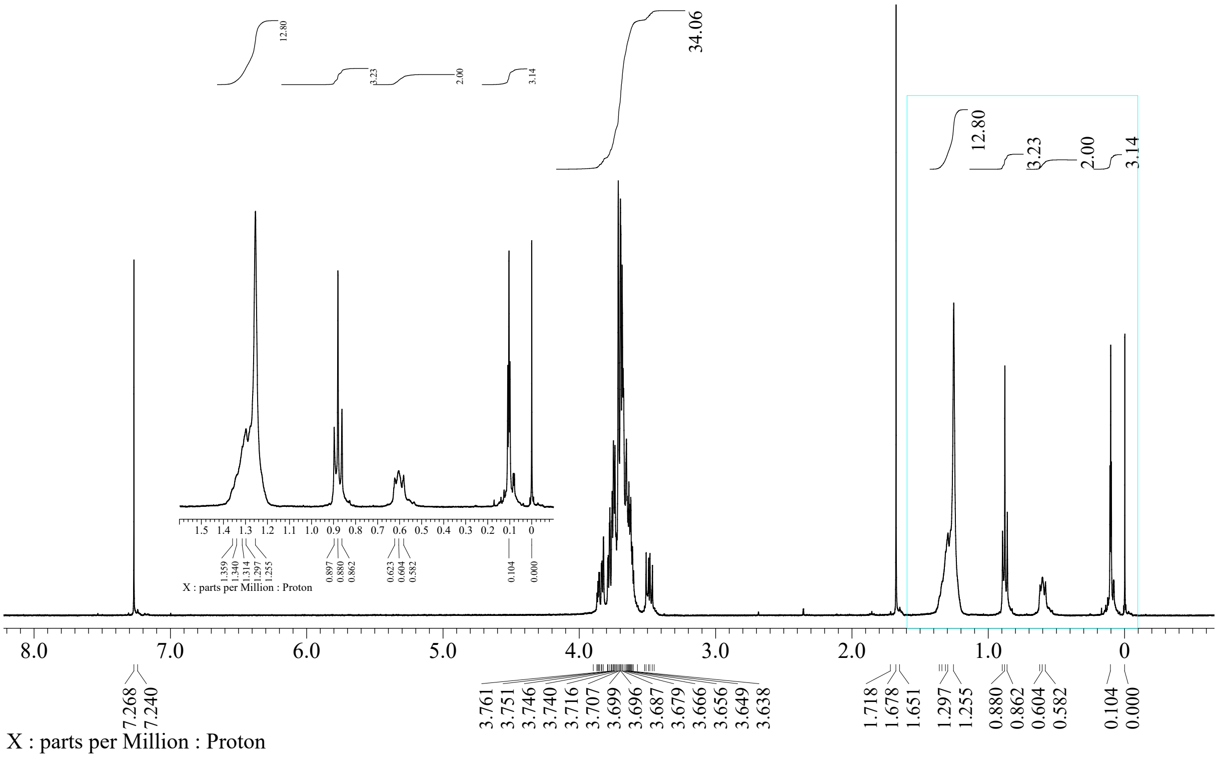

Figure S9. ^{1}H NMR spectrum of **7** (400 MHz; $CDCl_3$; Me_4Si).

The peaks at 1.68 ppm and 7.27 ppm are assigned to protons of H_2O and $CHCl_3$, respectively.

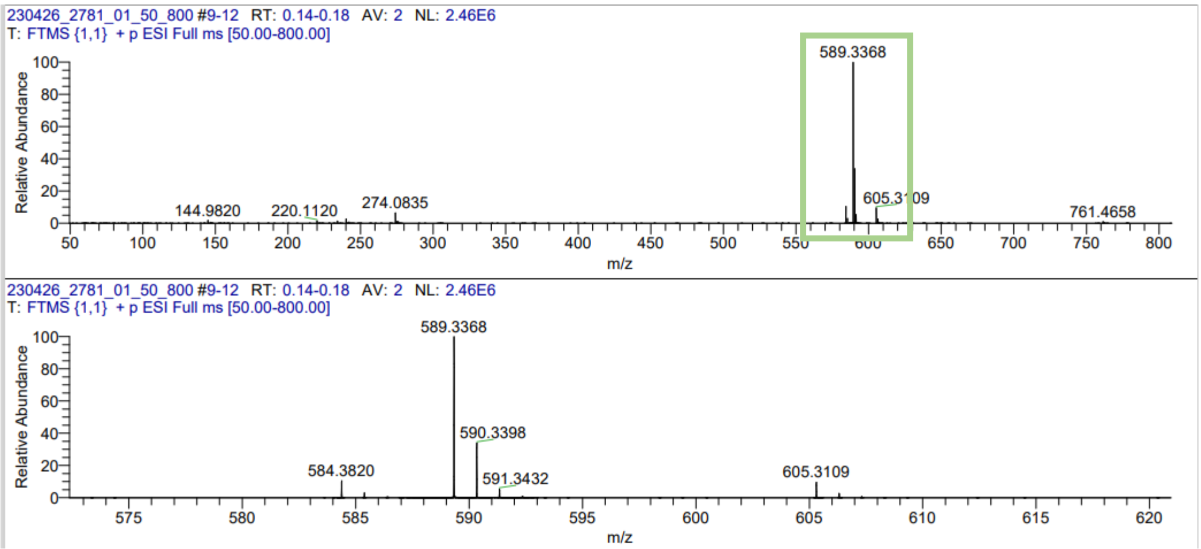

Figure S10. Mass spectrum of **7** (ESI+; CH_3CN).

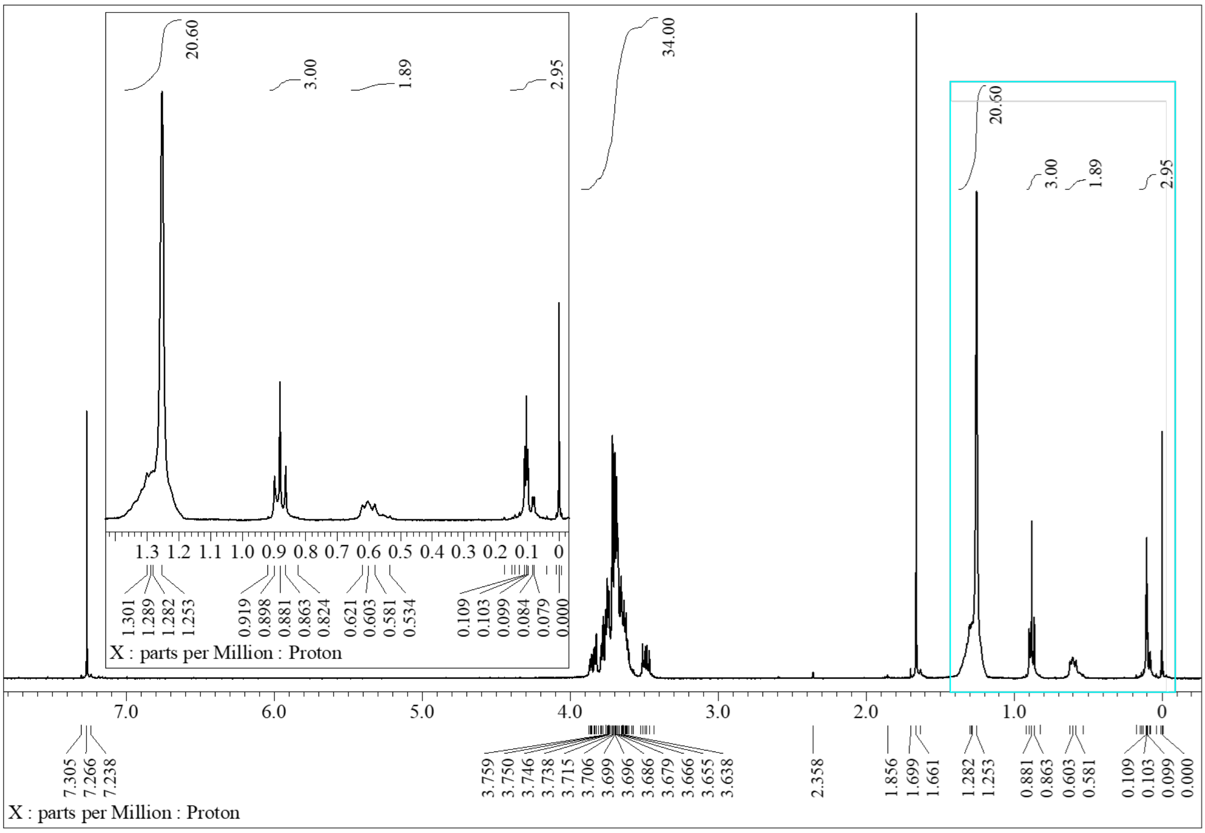

Figure S11. ^{1}H NMR spectrum of **8** (400 MHz; $CDCl_3$; Me_4Si).

The peaks at 1.66 ppm and 7.27 ppm are assigned to protons of H_2O and $CHCl_3$, respectively.

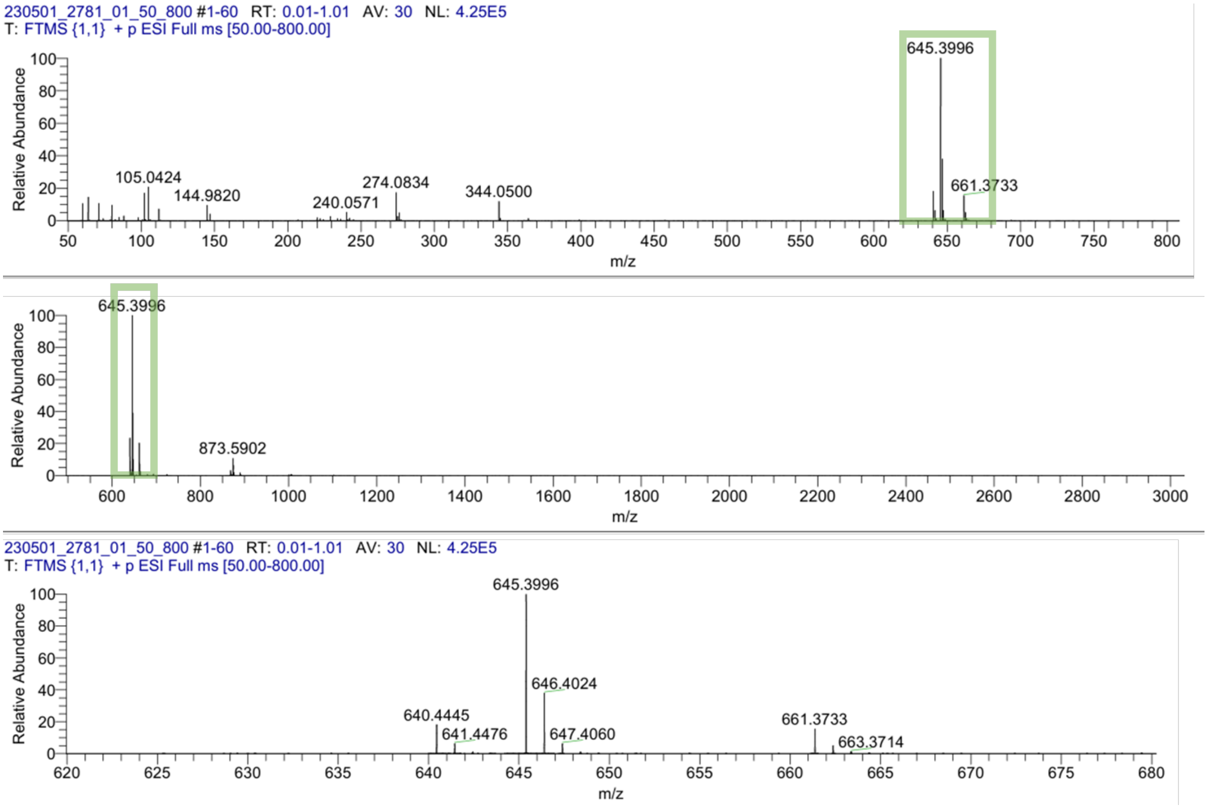

Figure S12. Mass spectrum of **8** (ESI+; CH_3CN).

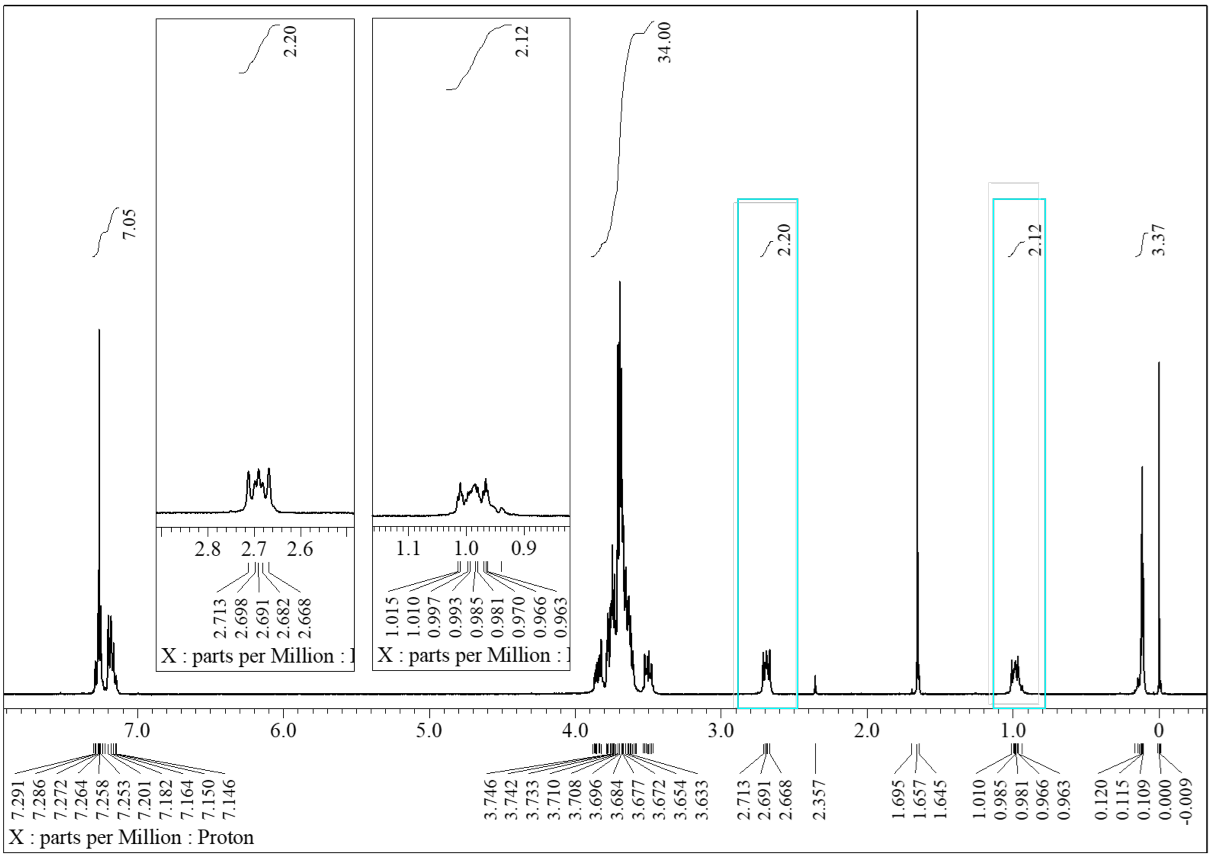

Figure S13. ^{1}H NMR spectrum of **9** (400 MHz; $CDCl_3$; Me_4Si).

The peaks at 1.66 ppm and 7.27 ppm are assigned to protons of H_2O and $CHCl_3$, respectively.

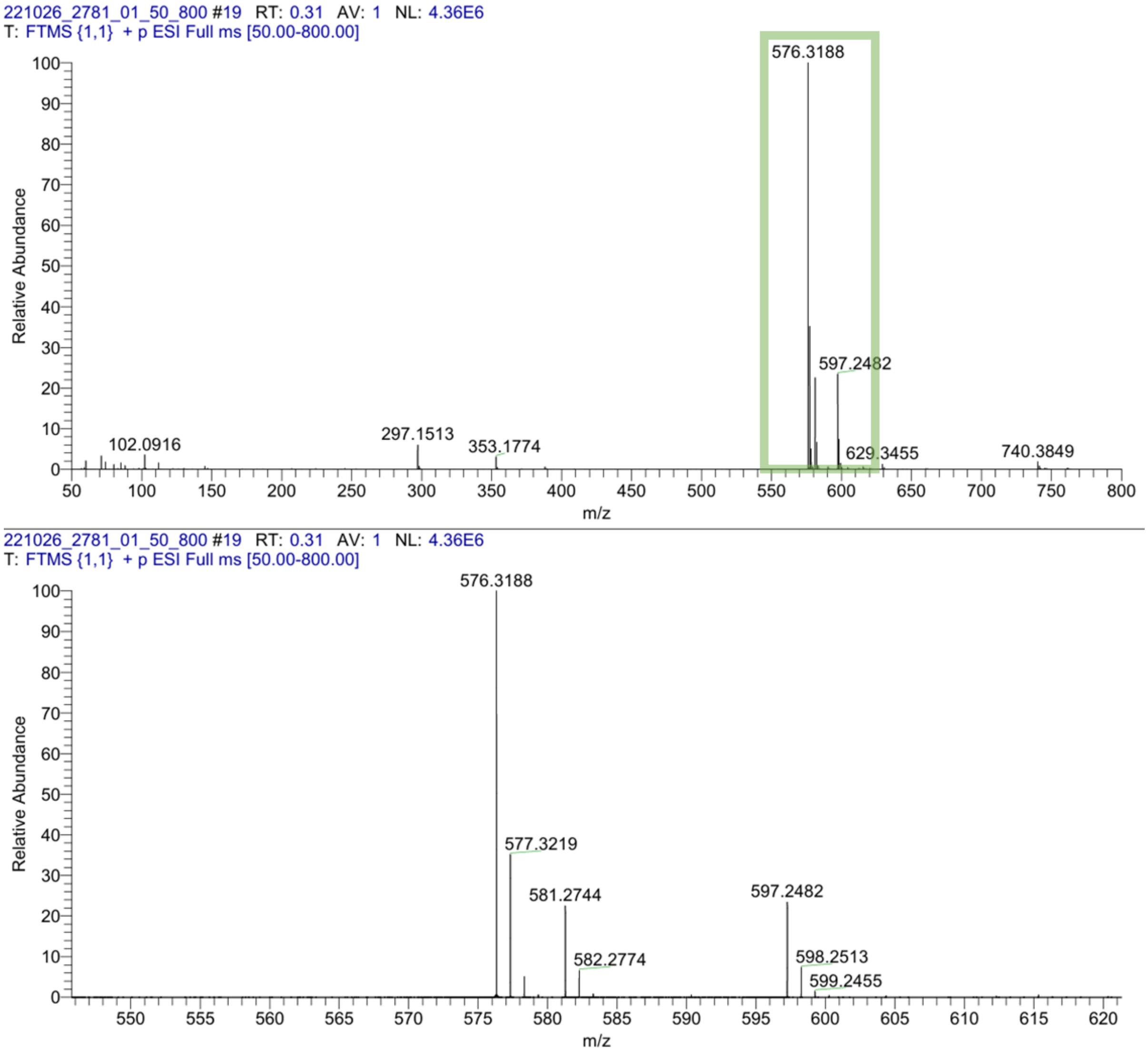

Figure S14. Mass spectrum of **9** (ESI+; CH_3CN).

Table S1. Emf (E) data of ion-selective electrodes measured at 25 °C

System: **1**-NaCl	
log a_{Na}	E (mV)
-6.00	-242.70
-5.00	-237.20
-4.01	-208.30
-3.02	-152.10
-2.05	-93.77
-1.12	-36.20

System: **1**-NaCl-LiCl*	
log a_{Na}	E (mV)
-6.09	-259.80
-5.09	-246.53
-4.09	-209.30
-3.09	-153.33
-2.10	-94.97
-1.13	-35.87

System: **1**-NaCl-KCl*	
log a_{Na}	E (mV)
-6.09	-154.40
-5.09	-154.90
-4.09	-151.40
-3.09	-130.90
-2.10	-84.33
-1.13	-27.73

System: **1**-NaCl-RbCl*	
log a_{Na}	E (mV)
-6.09	-210.30
-5.09	-211.40
-4.09	-205.00
-3.09	-173.70
-2.09	-120.90
-1.13	-62.77

System: **1**-NaCl-CsCl*	
log a_{Na}	E (mV)
-6.09	-200.10
-5.09	-200.00
-4.09	-190.00
-3.09	-150.10
-2.10	-95.03
-1.13	-37.37

System: **1**-NaCl-$MgCl_2$*	
log a_{Na}	E (mV)
-5.23	-235.00
-4.23	-212.00
-3.23	-155.00
-2.23	-96.00
-1.24	-37.40

System: **2**-NaCl-$CaCl_2$*	
log a_{Na}	E (mV)
-6.24	-231.10
-5.24	-228.10
-4.24	-206.40
-3.24	-156.20
-2.24	-99.03
-1.24	-39.53

System: **2**-NaCl-NH_4Cl*	
log a_{Na}	E (mV)
-6.19	-230.80
-5.19	-230.60
-4.19	-224.40
-3.19	-194.00
-2.19	-142.70
-1.19	-84.83

System: **2**-NaCl	
log a_{Na}	E (mV)
-6.00	-304.80
-5.00	-297.70
-4.00	-244.80
-3.01	-186.10
-2.04	-127.30
-1.11	-69.53

System: **2**-NaCl-LiCl*	
log a_{Na}	E (mV)
-6.09	-292.00
-5.09	-286.90
-4.09	-248.20
-3.09	-190.90
-2.09	-132.90
-1.13	-74.07

System: **2**-NaCl-KCl*	
log a_{Na}	E (mV)
-6.09	-263.60
-5.09	-259.60
-4.09	-252.40
-3.09	-211.90
-2.09	-155.90
-1.13	-96.07

System: **2**-NaCl-RbCl*	
log a_{Na}	E (mV)
-6.09	-246.40
-5.09	-246.30
-4.09	-221.30
-3.09	-166.70
-2.09	-108.30
-1.13	-49.50

System: **2**-NaCl-CsCl*	
log a_{Na}	E (mV)
-6.09	-298.00
-5.09	-278.20
-4.09	-228.60
-3.09	-170.10
-2.09	-111.10
-1.13	-50.83

System: **2**-NaCl-$MgCl_2$*	
log a_{Na}	E (mV)
-6.23	-323.60
-5.23	-299.10
-4.23	-248.90
-3.23	-191.40
-2.23	-131.40
-1.24	-73.20

System: **2**-NaCl-$CaCl_2$*	
log a_{Na}	E (mV)
-6.24	-269.50
-5.24	-265.50
-4.24	-241.40
-3.24	-191.00
-2.24	-133.90
-1.24	-75.57

System: **2**-NaCl-NH_4Cl*	
log a_{Na}	E (mV)
-6.19	-234.50
-5.19	-234.10
-4.19	-216.10
-3.19	-166.90
-2.19	-109.00
-1.19	-49.47

System: **2**-NaCl-HCl*	
log a_{Na}	E (mV)
-6.18	-304.90
-5.18	-296.50
-4.18	-267.70
-3.18	-213.60
-2.18	-155.00
-1.19	-95.90

System: **3**-NaCl	
log a_{Na}	E (mV)
-6.00	-297.80
-5.00	-299.00
-4.00	-250.00
-3.02	-196.70
-2.04	-137.30
-1.11	-76.97

System: **3**-NaCl-LiCl*	
log a_{Na}	E (mV)
-6.09	-325.80
-5.09	-312.60
-4.09	-274.60
-3.09	-223.90
-2.09	-170.60
-1.13	-117.80

System: **2**-NaCl-KCl*	
log a_{Na}	E (mV)
-6.09	-201.70
-5.09	-204.20
-4.09	-203.30
-3.09	-185.90
-2.09	-141.30
-1.13	-86.20

Table S1. Emf (E) data of ion-selective electrodes at 25 °C (continued)

System: **4**-NaCl	
log a_{Na}	E (mV)
-6.00	-268.50
-5.00	-266.30
-4.00	-224.20
-3.02	-165.70
-2.04	-107.30
-1.11	-49.30

System: **4**-NaCl-LiCl*	
log a_{Na}	E (mV)
-6.09	-291.60
-5.09	-289.60
-4.09	-248.90
-3.09	-192.00
-2.09	-133.40
-1.13	-75.70

System: **4**-NaCl-KCl*	
log a_{Na}	E (mV)
-6.09	-165.80
-5.09	-166.30
-4.09	-163.90
-3.09	-147.10
-2.09	-101.80
-1.13	-45.33

System: **4**-NaCl-RbCl*	
log a_{Na}	E (mV)
-6.09	-225.00
-5.09	-224.70
-4.09	-217.70
-3.09	-185.40
-2.09	-131.70
-1.13	-74.10

System: **4**-NaCl-CsCl*	
log a_{Na}	E (mV)
-6.09	-251.90
-5.09	-250.40
-4.09	-239.30
-3.09	-194.10
-2.09	-137.60
-1.13	-78.93

System: **4**-NaCl-$MgCl_2$*	
log a_{Na}	E (mV)
-5.23	-214.10
-4.23	-194.40
-3.23	-138.70
-2.23	-80.53
-1.24	-23.03

System: **4**-NaCl-$CaCl_2$*	
log a_{Na}	E (mV)
-6.23	-187.10
-5.23	-186.90
-4.23	-170.40
-2.23	-66.27
-1.24	-8.67

System: **4**-NaCl-NH_4Cl*	
log a_{Na}	E (mV)
-6.19	-236.10
-5.19	-237.70
-4.19	-230.60
-3.19	-198.80
-2.19	-144.60
-1.19	-85.83

System: **5**-NaCl	
log a_{Na}	E (mV)
-6.00	-283.80
-5.00	-279.20
-4.00	-227.70
-3.01	-170.50
-2.04	-112.30
-1.11	-54.70

System: **5**-NaCl-LiCl*	
log a_{Na}	E (mV)
-6.09	-295.90
-5.09	-286.70
-4.09	-245.60
-3.09	-188.70
-2.09	-130.20
-1.13	-73.03

System: **5**-NaCl-KCl*	
log a_{Na}	E (mV)
-6.09	-177.00
-5.09	-176.50
-4.09	-173.00
-3.09	-154.30
-2.09	-108.20
-1.13	-50.43

System: **5**-NaCl-RbCl*	
log a_{Na}	E (mV)
-6.09	-199.20
-5.09	-199.30
-4.09	-189.00
-3.09	-151.20
-2.09	-95.83
-1.13	-36.57

System: **5**-NaCl-CsCl*	
log a_{Na}	E (mV)
-6.09	-267.00
-5.09	-263.00
-4.09	-244.90
-3.09	-196.60
-2.09	-139.60
-1.13	-80.83

System: **5**-NaCl-$MgCl_2$*	
log a_{Na}	E (mV)
-6.23	-229.20
-5.23	-227.10
-4.23	-196.50
-3.23	-140.30
-2.23	-81.83
-1.24	-24.07

System: **5**-NaCl-$CaCl_2$*	
log a_{Na}	E (mV)
-6.23	-246.00
-5.23	-242.50
-4.23	-219.20
-2.23	-111.50
-1.24	-53.43

System: **5**-NaCl-NH_4Cl*	
log a_{Na}	E (mV)
-6.19	-245.70
-5.19	-245.20
-4.19	-235.50
-3.19	-196.70
-2.19	-141.90
-1.19	-83.60

System: **5**-NaCl-HCl*	
log a_{Na}	E (mV)
-5.18	-303.10
-4.18	-268.10
-3.18	-208.80
-2.18	-148.50
-1.19	-88.63

System: **6**-NaCl	
log a_{Na}	E (mV)
-6.00	-294.00
-5.00	-291.80
-4.00	-251.20
-3.02	-194.70
-2.04	-133.80
-1.11	-77.20

System: **6**-NaCl-KCl*	
log a_{Na}	E (mV)
-5.09	-188.60
-4.09	-189.40
-3.09	-182.50
-2.09	-139.60
-1.13	-87.77

Table S1. Emf (E) data of ion-selective electrodes at 25 °C (continued)

System: **8**-NaCl	
log a_{Na}	E (mV)
-6.00	-238.60
-5.00	-241.40
-4.00	-210.20
-3.01	-153.20
-2.04	-95.43
-1.11	-38.07

System: **8**-NaCl-KCl*	
log a_{Na}	E (mV)
-6.09	-160.80
-5.09	-161.30
-4.09	-158.90
-3.09	-142.50
-2.09	-97.70
-1.13	-40.73

System: **9**-NaCl	
log a_{Na}	E (mV)
-6.00	-296.50
-5.00	-285.10
-4.00	-256.90
-3.02	-196.80
-2.04	-138.70
-1.11	-83.13

System: **9**-NaCl-KCl*	
log a_{Na}	E (mV)
-193.00	-195.60
-196.40	-195.40
-197.50	-195.00
-196.30	-186.00
-194.60	-141.10
-195.30	-83.93

* Concentrations of coexisting chlorides: 0.050 mol/L for LiCl, KCl, RbCl, and CsCl; 0.47 mol/L for $MgCl_2$; 0.49 mol/L for $CaCl_2$ (**1** and **2**); 0.47 mol/L for $CaCl_2$ (**4** and **5**); 0.50 mol/L for NH_4Cl; 0.48 mol/L for HCl.

Table S2. Summary of the results of DFT calculations

[4-Na$^+$ complex]

Cartesian coordinates (angstroms), natural charges

Atom	No.	X	Y	Z	Natural charge
O	1	2.822636	-1.840401	1.430244	-0.62319
C	2	3.847951	-2.486589	2.180550	-0.11875
H	3	3.417292	-3.206971	2.890777	0.21944
H	4	4.432188	-3.034921	1.436627	0.24009
C	5	4.773673	-1.535658	2.936334	-0.12303
H	6	4.248070	-1.053221	3.772292	0.19741
H	7	5.603160	-2.120656	3.364258	0.22157
O	8	5.260845	-0.542152	2.042378	-0.61255
O	9	2.822449	1.840428	-1.429900	-0.62317
O	10	5.260507	0.542060	-2.042860	-0.61254
O	11	4.747716	-1.998563	-1.026878	-0.61819
O	12	2.380324	-0.831099	-1.932602	-0.61917
C	13	5.881566	0.558844	2.692314	-0.12524
H	14	5.302947	0.839260	3.583529	0.19780
H	15	6.897116	0.299091	3.030364	0.22232
C	16	5.967473	1.725943	1.709253	-0.12115
H	17	6.330174	2.620316	2.236275	0.22048
H	18	6.681337	1.484472	0.915517	0.23552
O	19	4.747511	1.998212	1.026116	-0.61818
C	20	3.749183	2.785099	1.673348	-0.12249
H	21	4.207600	3.615915	2.228811	0.21917
H	22	3.153366	3.198128	0.855479	0.23922
C	23	2.835114	1.994410	2.608878	-0.12300
H	24	3.360060	1.703855	3.530132	0.19644
H	25	1.990805	2.637194	2.904189	0.22231
O	26	2.380456	0.831031	1.932981	-0.61915
C	27	1.712547	-0.098693	2.781454	-0.12583
H	28	2.282185	-0.211387	3.714540	0.19838
H	29	0.705542	0.253320	3.034079	0.24253

C	30	1.601344	-1.453989	2.075595	0.06267
C	31	0.551011	-1.493089	0.940220	-0.10840
H	32	1.339849	-2.194063	2.846106	0.22857
C	33	3.847623	2.486596	-2.180399	-0.11874
H	34	3.416846	3.207074	-2.890457	0.21943
H	35	4.432044	3.034825	-1.436540	0.24009
C	36	4.773169	1.535731	-2.936499	-0.12303
H	37	5.602570	2.120820	-3.364479	0.22156
H	38	4.247407	1.053483	-3.772471	0.19740
C	39	5.881118	-0.558766	-2.693189	-0.12525
H	40	5.302256	-0.839031	-3.584292	0.19780
H	41	6.896553	-0.298885	-3.031483	0.22232
C	42	5.967416	-1.726059	-1.710408	-0.12114
H	43	6.330018	-2.620294	-2.237730	0.22049
H	44	6.681513	-1.484679	-0.916857	0.23552
C	45	3.749131	-2.785319	-1.673883	-0.12249
H	46	4.207332	-3.615992	-2.229733	0.21918
H	47	3.153676	-3.198563	-0.855858	0.23921
C	48	2.834607	-1.994449	-2.608812	-0.12301
H	49	1.990118	-2.637162	-2.903766	0.22231
H	50	3.359068	-1.703819	-3.530318	0.19645
C	51	1.712091	0.098637	-2.780827	-0.12585
H	52	2.281410	0.211337	-3.714101	0.19838
H	53	0.705004	-0.253393	-3.033098	0.24253
C	54	1.601023	1.453935	-2.074987	0.06266
C	55	0.550952	1.493083	-0.939386	-0.10840
H	56	1.339300	2.193979	-2.845446	0.22857
H	57	1.051410	-1.207885	0.009196	0.21594
O	58	-0.539813	-0.633887	1.225069	-0.93447
H	59	0.204963	-2.529678	0.822468	0.21441
H	60	0.205213	2.529751	-0.821388	0.21441
H	61	1.051399	1.207574	-0.008487	0.21595
O	62	-0.540165	0.634244	-1.224257	-0.93447
Si	63	-1.539603	-0.000054	0.000457	2.23831
C	64	-2.544366	1.333870	0.864479	-1.01760
H	65	-3.086896	0.864585	1.696936	0.26455

C	66	-2.543741	-1.334456	-0.863577	-1.01760
H	67	-3.085391	-0.865691	-1.696897	0.26456
H	68	-1.849420	-2.047068	-1.331327	0.25357
C	69	-3.530957	-2.087440	0.050278	-0.44556
H	70	-1.850417	2.046066	1.333403	0.25359
C	71	-3.530614	2.087580	-0.049825	-0.44556
H	72	-2.984250	2.539108	-0.890133	0.22858
C	73	-4.319569	3.179169	0.686497	-0.44649
H	74	-4.237477	1.375300	-0.498806	0.22836
H	75	-4.865187	2.724494	1.526282	0.22749
C	76	-5.305882	3.934343	-0.214283	-0.44854
H	77	-3.613190	3.893598	1.135108	0.22317
H	78	-6.010931	3.217909	-0.661187	0.22607
H	79	-4.758116	4.385535	-1.055134	0.22358
H	80	-2.985483	-2.538387	0.891481	0.22857
C	81	-4.319211	-3.179545	-0.686033	-0.44649
H	82	-4.238245	-1.374792	0.497998	0.22837
C	83	-5.306502	-3.933978	0.214295	-0.44854
C	84	-6.092572	-5.026648	-0.522743	-0.44785
H	85	-3.612408	-3.894359	-1.133363	0.22316
H	86	-4.863926	-2.725477	-1.526732	0.22749
H	87	-4.759630	-4.384618	1.056020	0.22358
H	88	-6.011949	-3.217166	0.659956	0.22607
H	89	-5.386689	-5.742824	-0.969352	0.22344
H	90	-6.639057	-4.575094	-1.363922	0.22569
C	91	-7.080464	-5.782374	0.375766	-0.45072
C	92	-6.092608	5.026553	0.522704	-0.44785
C	93	-7.079625	5.782887	-0.376251	-0.45072
H	94	-5.387134	5.742429	0.970441	0.22345
H	95	-6.639905	4.574493	1.363088	0.22569
H	96	-6.532911	6.235192	-1.217313	0.22315
C	97	-7.866301	6.875073	0.360626	-0.45127
H	98	-7.785684	5.067485	-0.823666	0.22472
C	99	-8.849350	7.625506	-0.544075	-0.67087
H	100	-7.160697	7.589871	0.807719	0.22515
H	101	-8.413120	6.422718	1.200143	0.22647

H	102	-8.328005	8.118257	-1.374080	0.22361
H	103	-9.394272	8.397062	0.011304	0.23382
H	104	-9.589767	6.942308	-0.978069	0.22462
H	105	-7.786933	-5.066661	0.822035	0.22472
H	106	-6.534552	-6.234131	1.217641	0.22315
C	107	-7.866441	-6.875039	-0.361139	-0.45127
H	108	-8.412221	-6.423294	-1.201660	0.22647
C	109	-8.850646	-7.624600	0.543031	-0.67087
H	110	-7.160418	-7.590301	-0.806826	0.22515
H	111	-9.591553	-6.940965	0.975497	0.22462
H	112	-8.330352	-8.116632	1.374123	0.22361
H	113	-9.394929	-8.396629	-0.012318	0.23382
Na	114	3.794289	-0.000340	-0.000099	0.86968

Thermochemistry

Temperature 298.150 Kelvin. Pressure 1.00000 Atm.
Zero-point correction= 1.023651 (Hartree/Particle)
Thermal correction to Energy= 1.078300
Thermal correction to Enthalpy= 1.079244
Thermal correction to Gibbs Free Energy= 0.927745
Sum of electronic and zero-point Energies= -2091.769439
Sum of electronic and thermal Energies= -2091.714790
Sum of electronic and thermal Enthalpies= -2091.713846
Sum of electronic and thermal Free Energies= -2091.865345

[**4**-K$^+$ complex]

Cartesian coordinates (angstroms), natural charges

Atom	No.	X	Y	Z	Natural charge
O	1	2.742909	-1.903237	1.643932	-0.61959
C	2	3.669681	-2.590999	2.477554	-0.11930
H	3	3.158238	-3.348803	3.089676	0.21948
H	4	4.336484	-3.112092	1.782568	0.23378
C	5	4.499748	-1.696591	3.399125	-0.12485
H	6	3.875681	-1.282116	4.202937	0.19759
H	7	5.276617	-2.314957	3.877145	0.22084
O	8	5.080613	-0.638233	2.648903	-0.60749
O	9	2.742877	1.903253	-1.644144	-0.61960
O	10	5.080694	0.638262	-2.648849	-0.60749
O	11	4.808989	-1.986708	-1.642752	-0.61662
O	12	2.278380	-0.826850	-2.128654	-0.61464
C	13	5.538378	0.439087	3.453799	-0.12475
H	14	4.780738	0.681170	4.212099	0.19598
H	15	6.461255	0.168656	3.992217	0.22036
C	16	5.834786	1.654273	2.572276	-0.12220
H	17	6.071547	2.512664	3.218453	0.22006
H	18	6.714968	1.447144	1.954643	0.23290
O	19	4.808819	1.986814	1.643073	-0.61662
C	20	3.700054	2.769851	2.076346	-0.12344
H	21	4.038654	3.621153	2.685653	0.21934
H	22	3.264416	3.162407	1.151988	0.23471
C	23	2.628222	1.999113	2.848651	-0.12414
H	24	2.987505	1.726235	3.850987	0.19635
H	25	1.754937	2.656902	2.986743	0.22040
O	26	2.278230	0.826794	2.128684	-0.61464
C	27	1.563771	-0.124288	2.911157	-0.12499
H	28	2.071909	-0.252096	3.876970	0.19584
H	29	0.540394	0.217052	3.108484	0.24018
C	30	1.488086	-1.472944	2.185390	0.06126
C	31	0.530599	-1.475125	0.972150	-0.10295

H	32	1.134325	-2.204961	2.927253	0.22757
C	33	3.669638	2.590969	-2.477816	-0.11930
H	34	3.158170	3.348655	-3.090061	0.21949
H	35	4.336374	3.112202	-1.782871	0.23378
C	36	4.499798	1.696488	-3.399234	-0.12485
H	37	5.276651	2.314840	-3.877296	0.22084
H	38	3.875792	1.281868	-4.203020	0.19759
C	39	5.538574	-0.439126	-3.453591	-0.12475
H	40	4.780995	-0.681340	-4.211911	0.19598
H	41	6.461469	-0.168701	-3.991980	0.22036
C	42	5.835002	-1.654195	-2.571913	-0.12220
H	43	6.071868	-2.512637	-3.217984	0.22006
H	44	6.715125	-1.446944	-1.954235	0.23291
C	45	3.700292	-2.769836	-2.076034	-0.12344
H	46	4.038978	-3.621170	-2.685248	0.21933
H	47	3.264607	-3.162330	-1.151673	0.23471
C	48	2.628485	-1.999214	-2.848490	-0.12414
H	49	1.755243	-2.657059	-2.986589	0.22040
H	50	2.987828	-1.726403	-3.850823	0.19635
C	51	1.563913	0.124121	-2.911254	-0.12499
H	52	2.072109	0.251880	-3.877043	0.19584
H	53	0.540570	-0.217293	-3.108617	0.24018
C	54	1.488099	1.472829	-2.185590	0.06126
C	55	0.530594	1.475049	-0.972351	-0.10295
H	56	1.134306	2.204766	-2.927516	0.22757
H	57	1.088819	-1.145584	0.088659	0.20910
O	58	-0.583467	-0.638024	1.223744	-0.93532
H	59	0.208279	-2.508773	0.783685	0.21477
H	60	0.208280	2.508703	-0.783914	0.21477
H	61	1.088797	1.145529	-0.088839	0.20909
O	62	-0.583487	0.637962	-1.223912	-0.93532
Si	63	-1.577368	-0.000021	-0.000070	2.23855
C	64	-2.582950	1.332676	0.865370	-1.01676
H	65	-3.129273	0.862691	1.694939	0.26403
C	66	-2.582996	-1.332701	-0.865483	-1.01676
H	67	-3.129334	-0.862706	-1.695038	0.26403

H	68	-1.888880	-2.042488	-1.337809	0.25344
C	69	-3.564301	-2.090008	0.051103	-0.44565
H	70	-1.888807	2.042450	1.337678	0.25344
C	71	-3.564267	2.090002	-0.051188	-0.44565
H	72	-3.013624	2.540826	-0.889075	0.22851
C	73	-4.353282	3.183242	0.682603	-0.44644
H	74	-4.271416	1.380010	-0.503379	0.22827
H	75	-4.903965	2.729652	1.519661	0.22744
C	76	-5.333275	3.941458	-0.222532	-0.44853
H	77	-3.646897	3.895498	1.134660	0.22313
H	78	-6.038223	3.227067	-0.672862	0.22603
H	79	-4.780190	4.391103	-1.060738	0.22349
H	80	-3.013641	-2.540841	0.888975	0.22851
C	81	-4.353356	-3.183236	-0.682663	-0.44644
H	82	-4.271424	-1.380003	0.503314	0.22827
C	83	-5.333333	-3.941435	0.222503	-0.44853
C	84	-6.120625	-5.035966	-0.510418	-0.44783
H	85	-3.646997	-3.895504	-1.134742	0.22313
H	86	-4.904058	-2.729638	-1.519705	0.22744
H	87	-4.780229	-4.391088	1.060692	0.22349
H	88	-6.038255	-3.227032	0.672855	0.22603
H	89	-5.415420	-5.749855	-0.961727	0.22345
H	90	-6.673683	-4.585609	-1.347931	0.22569
C	91	-7.100608	-5.794955	0.394001	-0.45071
C	92	-6.120526	5.036002	0.510416	-0.44783
C	93	-7.100527	5.795007	-0.393968	-0.45071
H	94	-5.415293	5.749879	0.961703	0.22345
H	95	-6.673563	4.585652	1.347948	0.22569
H	96	-6.547944	6.245455	-1.232194	0.22310
C	97	-7.888028	6.889744	0.338209	-0.45127
H	98	-7.806234	5.081522	-0.844991	0.22470
C	99	-8.863816	7.642694	-0.572256	-0.67087
H	100	-7.182818	7.602651	0.788923	0.22516
H	101	-8.440837	6.439313	1.174828	0.22648
H	102	-8.336381	8.133577	-1.399518	0.22359
H	103	-9.409576	8.416066	-0.020239	0.23383

H	104	-9.603785	6.961484	-1.010128	0.22461
H	105	-7.806288	-5.081458	0.845047	0.22470
H	106	-6.548003	-6.245411	1.232208	0.22310
C	107	-7.888152	-6.889680	-0.338148	-0.45127
H	108	-8.440983	-6.439240	-1.174749	0.22648
C	109	-8.863920	-7.642613	0.572351	-0.67087
H	110	-7.182969	-7.602599	-0.788887	0.22516
H	111	-9.603863	-6.961391	1.010249	0.22461
H	112	-8.336463	-8.133505	1.399596	0.22359
H	113	-9.409711	-8.415977	0.020355	0.23383
K	114	3.985855	0.000145	-0.000008	0.90720

Thermochemistry

Temperature 298.150 Kelvin. Pressure 1.00000 Atm.
Zero-point correction= 1.022403 (Hartree/Particle)
Thermal correction to Energy= 1.077626
Thermal correction to Enthalpy= 1.078570
Thermal correction to Gibbs Free Energy= 0.923666
Sum of electronic and zero-point Energies= -2119.696248
Sum of electronic and thermal Energies= -2119.641024
Sum of electronic and thermal Enthalpies= -2119.640080
Sum of electronic and thermal Free Energies= -2119.794985

Amounts of change from **4**-Na^+ complex to **4**-K^+ complex
Sum of electronic and zero-point Energies= -27.9268
Sum of electronic and thermal Energies= -27.9262
Sum of electronic and thermal Enthalpies= -27.9262
Sum of electronic and thermal Free Energies= -27.9296

[5-Na$^+$ complex]

Cartesian coordinates (angstroms), natural charges

Atom	No.	X	Y	Z	Natural charge
O	1	2.472438	-1.545346	1.738835	-0.62323
C	2	3.493429	-2.044034	2.599359	-0.11870
H	3	3.058719	-2.623127	3.426725	0.21942
H	4	4.080701	-2.718802	1.970604	0.23994
C	5	4.416702	-0.971521	3.173860	-0.12306
H	6	3.888488	-0.346169	3.907253	0.19741
H	7	5.244669	-1.469158	3.703246	0.22150
O	8	4.906961	-0.156172	2.116413	-0.61242
O	9	2.472518	1.545027	-1.739032	-0.62327
O	10	4.907447	0.156381	-2.116084	-0.61242
O	11	4.405545	-2.153708	-0.646979	-0.61807
O	12	2.036472	-1.172473	-1.743932	-0.61898
C	13	5.525746	1.044592	2.557860	-0.12520
H	14	4.942188	1.483933	3.378948	0.19773
H	15	6.538714	0.850405	2.944284	0.22223
C	16	5.620181	2.012019	1.378278	-0.12111
H	17	5.979509	2.987986	1.735488	0.22043
H	18	6.340254	1.629987	0.647868	0.23543
O	19	4.405748	2.154072	0.647406	-0.61809
C	20	3.403794	3.046079	1.132666	-0.12250
H	21	3.859177	3.966371	1.525908	0.21909
H	22	2.811439	3.298593	0.249618	0.23939
C	23	2.485407	2.441748	2.194394	-0.12300
H	24	3.005026	2.328629	3.156660	0.19641
H	25	1.638806	3.126637	2.360158	0.22221
O	26	2.036034	1.172375	1.743691	-0.61902
C	27	1.361538	0.415425	2.744661	-0.12574
H	28	1.926608	0.474201	3.685449	0.19831
H	29	0.354361	0.809774	2.923700	0.24205
C	30	1.249209	-1.045390	2.296953	0.06306
C	31	0.204706	-1.289325	1.182408	-0.10707

H	32	0.981588	-1.632439	3.187804	0.22839
C	33	3.493537	2.043732	-2.599556	-0.11871
H	34	3.058796	2.622660	-3.427012	0.21943
H	35	4.080722	2.718644	-1.970866	0.23994
C	36	4.416892	0.971181	-3.173801	-0.12305
H	37	5.244679	1.468773	-3.703514	0.22150
H	38	3.888671	0.345437	-3.906858	0.19741
C	39	5.526352	-1.044515	-2.557020	-0.12520
H	40	4.943004	-1.484111	-3.378122	0.19774
H	41	6.539421	-0.850427	-2.943220	0.22223
C	42	5.620401	-2.011568	-1.377116	-0.12111
H	43	5.980196	-2.987541	-1.733836	0.22042
H	44	6.339944	-1.629123	-0.646396	0.23542
C	45	3.404177	-3.046105	-1.132646	-0.12247
H	46	3.860078	-3.966096	-1.525992	0.21907
H	47	2.811667	-3.299103	-0.249842	0.23938
C	48	2.485926	-2.441839	-2.194551	-0.12299
H	49	1.639362	-3.126725	-2.360500	0.22220
H	50	3.005754	-2.328689	-3.156710	0.19639
C	51	1.361796	-0.415746	-2.744935	-0.12574
H	52	1.926814	-0.474509	-3.685761	0.19831
H	53	0.354660	-0.810247	-2.923868	0.24205
C	54	1.249324	1.045079	-2.297296	0.06307
C	55	0.204652	1.289028	-1.182898	-0.10708
H	56	0.981859	1.632133	-3.188182	0.22839
H	57	0.705354	-1.165370	0.217064	0.21564
O	58	-0.894852	-0.404512	1.312250	-0.93516
H	59	-0.131799	-2.333249	1.246117	0.21465
H	60	-0.132126	2.332844	-1.246892	0.21465
H	61	0.705257	1.165425	-0.217484	0.21564
O	62	-0.894658	0.403883	-1.312598	-0.93516
Si	63	-1.906949	-0.000126	-0.000194	2.23420
C	64	-2.923319	1.432847	0.684973	-1.02008
H	65	-3.351277	1.060272	1.626934	0.26877
C	66	-2.923832	-1.432724	-0.685365	-1.02008
H	67	-3.352141	-1.059819	-1.627033	0.26876

H	68	-2.243521	-2.240554	-0.987391	0.25445
C	69	-4.063028	-1.989075	0.206393	-0.23832
H	70	-2.242767	2.240670	0.986476	0.25444
C	71	-4.062796	1.989092	-0.206482	-0.23832
C	72	-3.533429	2.717668	-1.466480	-0.46154
C	73	-5.024908	2.849542	0.642438	-0.45075
H	74	-4.649408	1.131596	-0.571528	0.23480
H	75	-5.356999	2.242719	1.497206	0.23031
C	76	-6.265722	3.369071	-0.097415	-0.45380
H	77	-4.475436	3.697072	1.077122	0.22572
H	78	-6.776968	2.526951	-0.588076	0.22490
H	79	-5.968139	4.054733	-0.902772	0.22406
C	80	-3.533280	-2.718083	1.465982	-0.46155
C	81	-5.025567	-2.849175	-0.642402	-0.45075
H	82	-4.649392	-1.131605	0.571903	0.23479
C	83	-6.266175	-3.368737	0.097770	-0.45380
C	84	-7.257696	-4.090262	-0.826244	-0.45001
H	85	-4.476359	-3.696661	-1.077506	0.22572
H	86	-5.357897	-2.242088	-1.496890	0.23031
H	87	-5.968384	-4.054578	0.902898	0.22406
H	88	-6.777181	-2.526663	0.588761	0.22490
H	89	-6.744956	-4.924738	-1.325663	0.22615
H	90	-7.571347	-3.404437	-1.625968	0.22649
C	91	-8.493892	-4.617243	-0.090185	-0.67134
C	92	-7.256869	4.090857	0.826798	-0.45001
C	93	-8.493255	4.617830	0.091054	-0.67135
H	94	-6.743885	4.925370	1.325904	0.22615
H	95	-7.570322	3.405199	1.626744	0.22649
H	96	-8.214611	5.334799	-0.691018	0.22444
H	97	-9.046456	3.801929	-0.390107	0.22499
H	98	-9.046828	-3.801373	0.391338	0.22499
H	99	-8.215069	-5.334409	0.691639	0.22444
H	100	-9.179726	5.125207	0.777862	0.23539
H	101	-9.180674	-5.124370	-0.776867	0.23539
C	102	-2.803994	4.044842	-1.214214	-0.67725
H	103	-4.377503	2.906297	-2.140880	0.24017

H	104	-2.862761	2.039345	-2.010100	0.23330
H	105	-2.862348	-2.039997	2.009562	0.23332
C	106	-2.804071	-4.045254	1.213044	-0.67725
H	107	-4.377135	-2.906828	2.140624	0.24017
H	108	-1.942380	-3.925182	0.544641	0.21132
H	109	-3.463910	-4.795136	0.763555	0.23139
H	110	-2.432011	-4.462875	2.155610	0.23538
H	111	-3.463540	4.794856	-0.764516	0.23139
H	112	-2.432428	4.462246	-2.157071	0.23538
H	113	-1.941943	3.924855	-0.546262	0.21131
Na	114	3.452690	0.000452	0.000027	0.87000

Thermochemistry

Temperature 298.150 Kelvin. Pressure 1.00000 Atm.
Zero-point correction= 1.023906 (Hartree/Particle)
Thermal correction to Energy= 1.078094
Thermal correction to Enthalpy= 1.079038
Thermal correction to Gibbs Free Energy= 0.932527
Sum of electronic and zero-point Energies= -2091.759317
Sum of electronic and thermal Energies= -2091.705129
Sum of electronic and thermal Enthalpies= -2091.704185
Sum of electronic and thermal Free Energies= -2091.850695

[5-K+ complex]

Cartesian coordinates (angstroms), natural charges

Atom	No.	X	Y	Z	Natural charge
O	1	-2.397408	-1.350483	-2.116442	-0.61961
C	2	-3.319659	-1.771241	-3.116000	-0.11920
H	3	-2.804050	-2.319102	-3.918818	0.21944
H	4	-3.986512	-2.472282	-2.603117	0.23373
C	5	-4.150652	-0.651499	-3.743637	-0.12476
H	6	-3.525680	-0.020168	-4.390354	0.19750
H	7	-4.922825	-1.108198	-4.383651	0.22070
O	8	-4.739309	0.142900	-2.722486	-0.60745
O	9	-2.397224	1.350434	2.116879	-0.61961
O	10	-4.739298	-0.142831	2.722597	-0.60745
O	11	-4.472235	-2.371238	1.007520	-0.61660
O	12	-1.939902	-1.403650	1.802755	-0.61454
C	13	-5.202031	1.403988	-3.184466	-0.12473
H	14	-4.446880	1.855794	-3.842691	0.19599
H	15	-6.125350	1.295921	-3.776533	0.22031
C	16	-5.499692	2.314785	-1.991388	-0.12219
H	17	-5.741293	3.321253	-2.364366	0.22001
H	18	-6.377261	1.935983	-1.457068	0.23282
O	19	-4.471996	2.371652	-1.007913	-0.61660
C	20	-3.365893	3.248529	-1.202759	-0.12339
H	21	-3.707662	4.236315	-1.546358	0.21926
H	22	-2.929818	3.365056	-0.205481	0.23484
C	23	-2.293136	2.730568	-2.162036	-0.12401
H	24	-2.652769	2.751144	-3.200548	0.19618
H	25	-1.421687	3.403027	-2.108786	0.22027
O	26	-1.939759	1.403620	-1.802896	-0.61454
C	27	-1.216513	0.716380	-2.818611	-0.12490
H	28	-1.717376	0.868484	-3.784750	0.19579
H	29	-0.193002	1.101363	-2.902556	0.23976
C	30	-1.140945	-0.783067	-2.506452	0.06160
C	31	-0.188952	-1.131533	-1.340078	-0.10144

H	32	-0.781959	-1.273305	-3.424192	0.22736
C	33	-3.319381	1.771033	3.116591	-0.11920
H	34	-2.803666	2.318583	3.919552	0.21944
H	35	-3.986149	2.472326	2.603943	0.23373
C	36	-4.150513	0.651229	3.743940	-0.12476
H	37	-4.922613	1.107864	4.384088	0.22070
H	38	-3.525615	0.019646	4.390481	0.19750
C	39	-5.202204	-1.403965	3.184272	-0.12473
H	40	-4.447135	-1.856022	3.842417	0.19599
H	41	-6.125526	-1.295907	3.776338	0.22031
C	42	-5.499951	-2.314455	1.990980	-0.12219
H	43	-5.741697	-3.320974	2.363727	0.22001
H	44	-6.377454	-1.935416	1.456718	0.23282
C	45	-3.366230	-3.248273	1.202215	-0.12339
H	46	-3.708115	-4.236093	1.545603	0.21926
H	47	-2.930138	-3.364628	0.204927	0.23485
C	48	-2.293440	-2.730629	2.161627	-0.12401
H	49	-1.422067	-3.403176	2.108260	0.22027
H	50	-2.653093	-2.751366	3.200128	0.19618
C	51	-1.216525	-0.716704	2.818573	-0.12490
H	52	-1.717355	-0.868935	3.784709	0.19579
H	53	-0.193055	-1.101824	2.902391	0.23976
C	54	-1.140799	0.782792	2.506687	0.06160
C	55	-0.188856	1.131355	1.340305	-0.10143
H	56	-0.781677	1.272823	3.424484	0.22736
H	57	-0.747058	-1.057603	-0.400224	0.20869
O	58	0.932498	-0.267405	-1.347637	-0.93598
H	59	0.124789	-2.178711	-1.448512	0.21501
H	60	0.124899	2.178519	1.448825	0.21502
H	61	-0.746990	1.057517	0.400456	0.20868
O	62	0.932579	0.267211	1.347756	-0.93598
Si	63	1.939309	-0.000086	0.000026	2.23433
C	64	2.955721	1.495213	-0.535941	-1.01925
H	65	3.386566	1.219222	-1.509350	0.26831
C	66	2.955792	-1.495356	0.535940	-1.01925
H	67	3.386704	-1.219337	1.509311	0.26831

H	68	2.273511	-2.327383	0.757025	0.25434
C	69	4.092413	-1.962784	-0.408321	-0.23840
H	70	2.273402	2.327226	-0.756959	0.25434
C	71	4.092397	1.962649	0.408250	-0.23840
C	72	3.561162	2.551259	1.738685	-0.46147
C	73	5.047333	2.914295	-0.346351	-0.45067
H	74	4.685202	1.075369	0.680351	0.23485
H	75	5.381815	2.403385	-1.260807	0.23027
C	76	6.285906	3.361237	0.442946	-0.45381
H	77	4.491331	3.799237	-0.688340	0.22555
H	78	6.803728	2.475433	0.841072	0.22490
H	79	5.984948	3.955560	1.316721	0.22404
C	80	3.561101	-2.551551	-1.738658	-0.46147
C	81	5.047492	-2.914289	0.346280	-0.45067
H	82	4.685127	-1.075481	-0.680546	0.23485
C	83	6.286091	-3.361113	-0.443043	-0.45381
C	84	7.270905	-4.183595	0.400240	-0.44999
H	85	4.491608	-3.799281	0.688330	0.22555
H	86	5.381948	-2.403299	1.260700	0.23026
H	87	5.985174	-3.955517	-1.316777	0.22404
H	88	6.803787	-2.475264	-0.841233	0.22490
H	89	6.751716	-5.063089	0.807196	0.22610
H	90	7.587431	-3.588999	1.268898	0.22646
C	91	8.505107	-4.637442	-0.386176	-0.67134
C	92	7.270584	4.183884	-0.400335	-0.44999
C	93	8.504761	4.637844	0.386055	-0.67134
H	94	6.751270	5.063335	-0.807225	0.22610
H	95	7.587149	3.589372	-1.269036	0.22646
H	96	8.222785	5.265877	1.240101	0.22443
H	97	9.063922	3.779079	0.777349	0.22499
H	98	9.064145	-3.778628	-0.777537	0.22499
H	99	8.223174	-5.265554	-1.240179	0.22443
H	100	9.186901	5.219754	-0.243590	0.23537
H	101	9.187344	-5.219235	0.243471	0.23537
C	102	2.817406	3.889827	1.628190	-0.67705
H	103	4.406305	2.677429	2.426205	0.23994

H	104	2.899303	1.813776	2.211219	0.23323
H	105	2.899218	-1.814123	-2.211242	0.23323
C	106	2.817337	-3.890097	-1.627955	-0.67705
H	107	4.406203	-2.677808	-2.426213	0.23994
H	108	1.951936	-3.830027	-0.956317	0.21143
H	109	3.466500	-4.689110	-1.253845	0.23121
H	110	2.448059	-4.204750	-2.610765	0.23524
H	111	3.466556	4.688877	1.254135	0.23121
H	112	2.448195	4.204366	2.611061	0.23524
H	113	1.951963	3.829849	0.956600	0.21143
K	114	-3.645357	0.000301	0.000123	0.90730

Thermochemistry

Temperature 298.150 Kelvin. Pressure 1.00000 Atm.
Zero-point correction= 1.022644 (Hartree/Particle)
Thermal correction to Energy= 1.077428
Thermal correction to Enthalpy= 1.078372
Thermal correction to Gibbs Free Energy= 0.928269
Sum of electronic and zero-point Energies= -2119.686194
Sum of electronic and thermal Energies= -2119.631410
Sum of electronic and thermal Enthalpies= -2119.630466
Sum of electronic and thermal Free Energies= -2119.780569

Amounts of change from **5**-Na^+ complex to **5**-K^+ complex
Sum of electronic and zero-point Energies= -27.9269
Sum of electronic and thermal Energies= -27.9263
Sum of electronic and thermal Enthalpies= -27.9263
Sum of electronic and thermal Free Energies= -27.9299

[6-Na$^+$ complex]

Cartesian coordinates (angstroms), natural charges

Atom	No.	X	Y	Z	Natural charge
O	1	1.370896	0.084796	-2.334378	-0.62266
C	2	2.397291	-0.080101	-3.309803	-0.11879
H	3	1.968057	-0.157307	-4.319093	0.21935
H	4	2.986556	0.838858	-3.249250	0.24036
C	5	3.315405	-1.276007	-3.066546	-0.12308
H	6	2.784415	-2.223099	-3.236502	0.19738
H	7	4.146498	-1.231992	-3.788199	0.22162
O	8	3.800224	-1.233897	-1.730049	-0.61235
O	9	1.371194	-0.085727	2.334492	-0.62265
O	10	3.800325	1.233397	1.729971	-0.61235
O	11	3.288930	2.072251	-0.876036	-0.61859
O	12	0.925484	2.004370	0.600778	-0.61936
C	13	4.407195	-2.445701	-1.302405	-0.12519
H	14	3.815272	-3.302346	-1.653826	0.19777
H	15	5.419353	-2.552783	-1.723369	0.22221
C	16	4.501275	-2.437829	0.222960	-0.12106
H	17	4.854217	-3.419382	0.571071	0.22049
H	18	5.226328	-1.682112	0.541043	0.23545
O	19	3.289097	-2.072744	0.876016	-0.61859
C	20	2.281175	-3.062977	1.074293	-0.12265
H	21	2.731144	-4.024612	1.360077	0.21919
H	22	1.692039	-2.689092	1.915304	0.23968
C	23	1.361010	-3.272515	-0.128041	-0.12378
H	24	1.876113	-3.809431	-0.937472	0.19613
H	25	0.506922	-3.893581	0.184106	0.22477
O	26	0.925479	-2.005392	-0.600548	-0.61934
C	27	0.257659	-2.065177	-1.857357	-0.12556
H	28	0.826465	-2.712890	-2.539214	0.19818
H	29	-0.749186	-2.484162	-1.746134	0.24224
C	30	0.149006	-0.656700	-2.451451	0.06238
C	31	-0.899653	0.244812	-1.759178	-0.10859

H	32	-0.110727	-0.777040	-3.513545	0.22812
Na	33	2.345867	-0.000250	-0.000013	0.87012
C	34	2.397714	0.079247	3.309774	-0.11879
H	35	1.968620	0.156277	4.319137	0.21935
H	36	2.987128	-0.839603	3.249031	0.24036
C	37	3.315567	1.275343	3.066502	-0.12308
H	38	4.146714	1.231475	3.788100	0.22162
H	39	2.784387	2.222317	3.236529	0.19738
C	40	4.407021	2.445355	1.302375	-0.12519
H	41	3.814940	3.301851	1.653893	0.19777
H	42	5.419178	2.552621	1.723292	0.22221
C	43	4.501030	2.437634	-0.222999	-0.12106
H	44	4.853673	3.419319	-0.571041	0.22049
H	45	5.226289	1.682157	-0.541180	0.23545
C	46	2.280734	3.062226	-1.074252	-0.12265
H	47	2.730435	4.023959	-1.360127	0.21920
H	48	1.691610	2.688154	-1.915188	0.23968
C	49	1.360631	3.271590	0.128160	-0.12379
H	50	0.506344	3.892410	-0.183928	0.22477
H	51	1.875673	3.808678	0.937514	0.19614
C	52	0.257600	2.064096	1.857570	-0.12557
H	53	0.826216	2.712036	2.539367	0.19819
H	54	-0.749348	2.482814	1.746261	0.24222
C	55	0.149256	0.655638	2.451790	0.06237
C	56	-0.899448	-0.246067	1.759869	-0.10856
H	57	-0.110290	0.776029	3.513924	0.22812
H	58	-0.402692	0.770613	-0.937987	0.21752
O	59	-1.997096	-0.522867	-1.288165	-0.93339
H	60	-1.242445	1.004158	-2.474403	0.21980
H	61	-1.241204	-1.005915	2.475067	0.21979
H	62	-0.402929	-0.771255	0.938028	0.21752
O	63	-1.997728	0.521216	1.290221	-0.93342
Si	64	-2.966711	-0.000124	0.000489	2.22763
C	65	-3.938648	-1.521743	0.488168	-0.59330
C	66	-3.935971	1.522919	-0.488224	-0.59329
C	67	-5.456740	3.766708	-1.242282	-0.21397

C	68	-5.184164	2.762729	-2.174161	-0.22994
C	69	-4.432048	1.649106	-1.797755	-0.20865
C	70	-4.220770	2.541640	0.437261	-0.20462
C	71	-4.974569	3.655392	0.063859	-0.23131
H	72	-6.046012	4.632300	-1.532545	0.24348
H	73	-5.559476	2.846628	-3.190446	0.24278
H	74	-4.227282	0.873023	-2.532229	0.23685
H	75	-3.847344	2.464562	1.455011	0.24038
H	76	-5.188107	4.434712	0.790536	0.24179
C	77	-5.463470	-3.763252	1.240746	-0.21396
C	78	-4.981490	-3.651735	-0.065452	-0.23131
C	79	-4.225683	-2.539106	-0.438122	-0.20459
C	80	-4.434506	-1.648126	1.797758	-0.20862
C	81	-5.188658	-2.760624	2.173421	-0.22995
H	82	-6.054310	-4.627965	1.530439	0.24348
H	83	-5.196721	-4.430030	-0.792726	0.24179
H	84	-3.852358	-2.461919	-1.455904	0.24036
H	85	-4.227924	-0.873101	2.532832	0.23687
H	86	-5.563785	-2.844710	3.189759	0.24278

Thermochemistry

Temperature 298.150 Kelvin. Pressure 1.00000 Atm.
Zero-point correction= 0.730685 (Hartree/Particle)
Thermal correction to Energy= 0.772296
Thermal correction to Enthalpy= 0.773240
Thermal correction to Gibbs Free Energy= 0.655596
Sum of electronic and zero-point Energies= -1925.143336
Sum of electronic and thermal Energies= -1925.101726
Sum of electronic and thermal Enthalpies= -1925.100781
Sum of electronic and thermal Free Energies= -1925.218426

[**6**-K$^+$ complex]

Cartesian coordinates (angstroms), natural charges

Atom	No.	X	Y	Z	Natural charge
O	1	1.332033	-0.650800	-2.428581	-0.61928
C	2	2.262082	-1.106897	-3.405434	-0.11939
H	3	1.755333	-1.322269	-4.358000	0.21948
H	4	2.935692	-0.258556	-3.565743	0.23395
C	5	3.080562	-2.332883	-2.998578	-0.12501
H	6	2.449507	-3.232198	-2.982506	0.19769
H	7	3.861481	-2.497893	-3.758379	0.22104
O	8	3.654224	-2.126024	-1.714631	-0.60758
O	9	1.331815	0.650919	2.428836	-0.61929
O	10	3.653777	2.126402	1.714736	-0.60759
O	11	3.373991	2.345827	-1.089441	-0.61696
O	12	0.853617	2.257060	0.175790	-0.61486
C	13	4.083306	-3.326838	-1.088223	-0.12464
H	14	3.307345	-4.097235	-1.197665	0.19598
H	15	4.997914	-3.717603	-1.563134	0.22022
C	16	4.385005	-3.062565	0.388539	-0.12221
H	17	4.600650	-4.021119	0.883620	0.22004
H	18	5.279374	-2.435756	0.468174	0.23295
O	19	3.374509	-2.345734	1.089495	-0.61697
C	20	2.253415	-3.051080	1.615577	-0.12362
H	21	2.576856	-3.967673	2.131138	0.21939
H	22	1.827892	-2.371283	2.360518	0.23504
C	23	1.177363	-3.409200	0.589410	-0.12516
H	24	1.523364	-4.212017	-0.076950	0.19608
H	25	0.293574	-3.790948	1.124913	0.22348
O	26	0.854035	-2.257199	-0.175682	-0.61486
C	27	0.144213	-2.556383	-1.372416	-0.12464
H	28	0.653009	-3.375517	-1.899449	0.19563
H	29	-0.879939	-2.879832	-1.150768	0.23990
C	30	0.075112	-1.322049	-2.280145	0.06102
C	31	-0.881632	-0.223258	-1.765994	-0.10334

H	32	-0.273953	-1.676361	-3.262061	0.22701
K	33	2.581798	0.000011	0.000271	0.90770
C	34	2.261825	1.107178	3.405653	-0.11939
H	35	1.755068	1.322548	4.358217	0.21948
H	36	2.935529	0.258921	3.566008	0.23395
C	37	3.080178	2.333228	2.998721	-0.12501
H	38	3.861133	2.498328	3.758466	0.22104
H	39	2.449039	3.232481	2.982677	0.19770
C	40	4.082555	3.327267	1.088211	-0.12464
H	41	3.306402	4.097475	1.197594	0.19598
H	42	4.997068	3.718305	1.563083	0.22022
C	43	4.384306	3.062961	-0.388536	-0.12221
H	44	4.599717	4.021538	-0.883677	0.22004
H	45	5.278834	2.436373	-0.468120	0.23295
C	46	2.252764	3.050875	-1.615649	-0.12362
H	47	2.576042	3.967424	-2.131390	0.21939
H	48	1.827359	2.370851	-2.360446	0.23504
C	49	1.176652	3.408984	-0.589541	-0.12516
H	50	0.292760	3.790419	-1.125096	0.22348
H	51	1.522486	4.212021	0.076640	0.19608
C	52	0.143790	2.556292	1.372509	-0.12465
H	53	0.652514	3.375524	1.899457	0.19563
H	54	-0.880398	2.879622	1.150848	0.23990
C	55	0.074819	1.322025	2.280335	0.06102
C	56	-0.881821	0.223094	1.766263	-0.10333
H	57	-0.274290	1.676375	3.262221	0.22701
H	58	-0.329479	0.424422	-1.076419	0.21118
O	59	-2.005938	-0.811398	-1.132388	-0.93430
H	60	-1.196273	0.400019	-2.613382	0.22003
H	61	-1.196365	-0.400185	2.613688	0.22004
H	62	-0.329619	-0.424556	1.076697	0.21117
O	63	-2.006205	0.811060	1.132662	-0.93431
Si	64	-2.967860	-0.000148	0.000003	2.22789
C	65	-3.939362	-1.363557	0.834748	-0.59155
C	66	-3.939040	1.363356	-0.834966	-0.59155
C	67	-5.460694	3.363024	-2.101117	-0.21426

C	68	-5.155971	2.181791	-2.781034	-0.23004
C	69	-4.403539	1.189783	-2.150506	-0.20905
C	70	-4.256512	2.557242	-0.164684	-0.20483
C	71	-5.010484	3.550063	-0.792149	-0.23133
H	72	-6.050187	4.134545	-2.588968	0.24341
H	73	-5.506438	2.033475	-3.798848	0.24261
H	74	-4.173240	0.273440	-2.689808	0.23669
H	75	-3.908550	2.712901	0.853188	0.23972
H	76	-5.249255	4.467575	-0.260946	0.24170
C	77	-5.461493	-3.363083	2.100543	-0.21426
C	78	-5.010261	-3.550531	0.791988	-0.23133
C	79	-4.256049	-2.557779	0.164698	-0.20483
C	80	-4.404880	-1.189578	2.149877	-0.20903
C	81	-5.157552	-2.181512	2.780227	-0.23004
H	82	-6.051174	-4.134548	2.588257	0.24341
H	83	-5.248423	-4.468304	0.260964	0.24170
H	84	-3.907317	-2.713723	-0.852866	0.23972
H	85	-4.175168	-0.272993	2.689018	0.23669
H	86	-5.508816	-2.032880	3.797720	0.24261

Thermochemistry

Temperature 298.150 Kelvin. Pressure 1.00000 Atm.
Zero-point correction= 0.729499 (Hartree/Particle)
Thermal correction to Energy= 0.771658
Thermal correction to Enthalpy= 0.772602
Thermal correction to Gibbs Free Energy= 0.651713
Sum of electronic and zero-point Energies= -1953.069961
Sum of electronic and thermal Energies= -1953.027802
Sum of electronic and thermal Enthalpies= -1953.026857
Sum of electronic and thermal Free Energies= -1953.147746

Amounts of change from **6**-Na^+ complex to **6**-K^+ complex
Sum of electronic and zero-point Energies= -27.9266
Sum of electronic and thermal Energies= -27.9261
Sum of electronic and thermal Enthalpies= -27.9261
Sum of electronic and thermal Free Energies= -27.9293
